# Statistically Designed Medium Reveals Interactions between Metabolism and Genetic Information Processing for Production of Stable Human Serum Albumin in *Pichia pastoris*

**DOI:** 10.3390/biom9100568

**Published:** 2019-10-04

**Authors:** Nitu Maity, Saroj Mishra

**Affiliations:** Department of Biochemical Engineering and Biotechnology, Indian Institute of Technology Delhi, Hauz-Khas, New-Delhi 110016, India; bez158502@dbeb.iitd.ac.in

**Keywords:** *Pichia pastoris*, human serum albumin, central composite design, transcriptome analysis

## Abstract

Human serum albumin (HSA), sourced from human serum, has been an important therapeutic protein for several decades. *Pichia pastoris* is strongly considered as an expression platform, but proteolytic degradation of recombinant HSA in the culture filtrate remains a major bottleneck for use of this system. In this study, we have reported the development of a medium that minimized proteolytic degradation across different copy number constructs. A synthetic codon-optimized copy of HSA was cloned downstream of α–factor secretory signal sequence and expressed in *P. pastoris* under the control of Alcohol oxidase 1 promoter. A two-copy expression cassette was also prepared. Culture conditions and medium components were identified and optimized using statistical tools to develop a medium that supported stable production of HSA. Comparative analysis of transcriptome data obtained by cultivation on optimized and unoptimized medium indicated upregulation of genes involved in methanol metabolism, alternate nitrogen assimilation, and DNA transcription, whereas enzymes of translation and secretion were downregulated. Several new genes were identified that could serve as possible targets for strain engineering of this yeast.

## 1. Introduction

Since the 1970s, an exponential rise in commercial production of pharmaceuticals has led to the development of several biotechnology industries. More than 25% of these are comprised of biopharmaceuticals which are largely produced through recombinant DNA approach [1]. The methylotrophic yeast *Pichia pastoris* is considered an efficient expression platform for production of human therapeutics [2,3]. In spite of a large body of information available on the genetics of the yeast, little is known about how external fermentation conditions affect cell physiology and the network of metabolism and genetic information processing. Since methanol possesses higher enthalpy consumption (−727 kJC/mol) in comparison to sugars (glycerol: −549 kJC/mol), it leads to heat generation. Also, rapid methanol metabolism is accompanied by the accumulation of formaldehyde, leading to cellular toxicity. Although this can be partially alleviated by combining a second sugar, such as lactose [4], sorbitol [5], or glycerol [6] in the production phase, the regulation of methanol feed remains critical in *Pichia* fermentation. Temperature is also considered to be important, as high temperature leads to accumulation of improperly folded proteins triggering stress pathways that lead to cell death [7].

The quality of the fermentation broth containing the end-product is of prime concern [8], as the final cost of the product will be determined by downstream processing steps. Human serum albumin (HSA), which is currently sourced from human serum, is an important therapeutic administered in trauma, injury, hypoalbuminemia, and hypoproteinemia [9,10]. Recombinant HSA has been produced in *Hansenula polymorpha* [11], *Kluyveromyces lactis* [12], *Saccharomyces cerevisiae* [13], rice [14], cattle [15], and mammalian cell lines [16]. Among the microbial systems, *P. pastoris* is considered to be the most promising platform [17]. One of the major challenges in this system has been the presence of contaminating proteins and instability of the secreted HSA. Different cytosolic peptidases such as prosome, multi-protease complex, multi-catalytic protease, proteasome, and vacuolar proteases, e.g., aminopeptidase Y, carboxypeptidase Y and C, and proteinase A and B, have been reported to accelerate the protein degradation process [18]. Different strategies have been adopted to slow down proteolysis using protease deficient strains, supplying mixed carbon sources during the production phase and lowering of peptone levels [19].

In general, the optimum conditions for production of a protein are identified by studying one parameter at a time [20,21] until the optimum is identified. However, this approach is time-consuming and does not lead to identification of interdependence of parameters which could be crucial for production. Also, such approach does not ensure the stability of the protein. The result would thus fail to arrive at the optimal conditions. Design of experiments (DOE) methodology has been successfully used by several researchers to address these issues during optimization studies [22,23,24]. With DOE, interactions between several factors can be identified and a more reliable set of conditions can be arrived at. This also follows a more systematic approach toward experimental setup and reasonably cuts down on the number of experiments to be conducted. In this study, a novel approach was undertaken to arrive at conditions conducive to stable production of HSA in the culture supernatant by monitoring the level of HSA in the gel by gel densitometry method. The optimized cultivation conditions were applied and the underlying cellular networks were explored by way of comparative transcriptome analysis under the optimized and the unoptimized conditions. The overall layout of the work consisted of (i) identification of key parameters that affected stable production of HSA, which were temperature, methanol concentration and its delivery, and peptone concentration; (ii) investigating the effect of these parameters alone and in combination using response surface methodology (RSM) [25] with an objective to arrive at conditions leading to stable production of HSA; (iii) investigating differential gene expression on the unoptimized and optimized medium to identify key up- and down-regulated genes; and (iv) map the affected genes under carbon and nitrogen metabolism, DNA replication, translation, folding, and secretion. The long-term goal is to identify genes whose expression can be modified to produce engineered yeast suitable for production of foreign proteins.

## 2. Materials and Methods

### 2.1. Materials

All chemicals, unless otherwise described, were procured from local companies or Merck Ltd., Mumbai, India. These were used directly without any further purification.

### 2.2. Strains and Construction of Single- and 2-Copy Expression Cassettes of HSA

*Escherichia coli* DH5α was used for molecular cloning work and large-scale preparation of the vector. It was maintained and cultivated on Luria-Bertani (LB) (HiMedia Labs, Delhi, India). Zeocin (Invitrogen, Thermo Fisher Scientific, USA) was added (100 mg/L) to LB for propagation of the vector. *P. pastoris* X-33 (Invitrogen) was used as the expression host and maintained on YPD (HiMedia).

**Figure 1:**
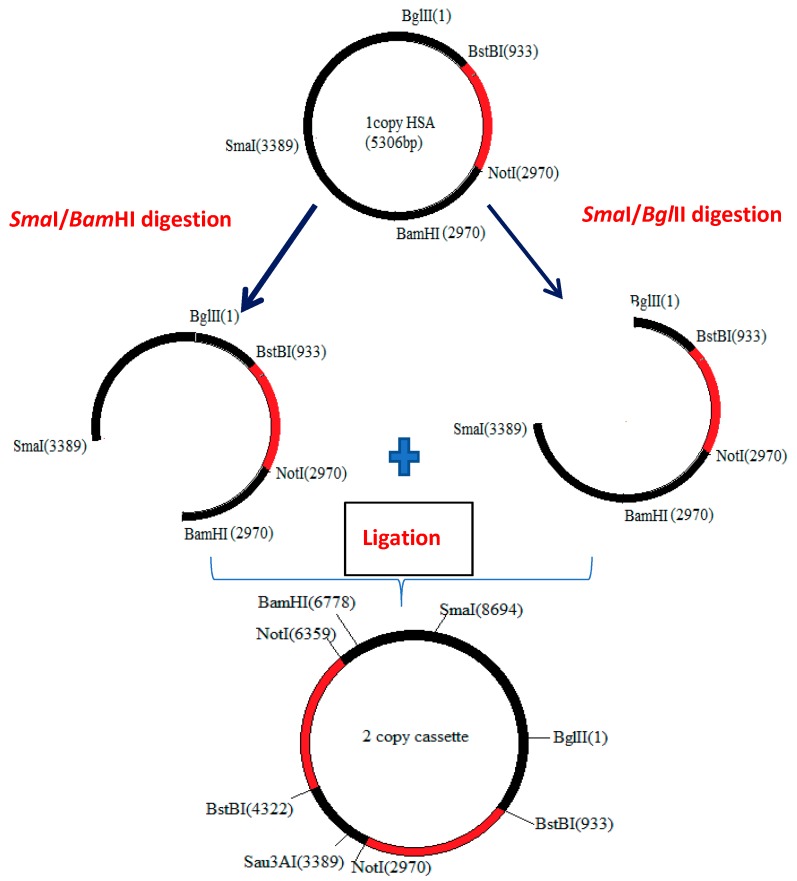
Scheme for construction of the two-copy human serum albumin (HSA) vector (the red region indicates HSA expression cassette). The sizes shown are not to actual scale.

A codon optimized copy of the human HSA (CO-HSA) (Genbank NCBI Accession No. KC416181), containing codon optimized α-mating secretory sequence and two stop codons (TGA and TAA in tandem) was designed according to the highest expressed genes in *P. pastoris* [26]. The synthetic HSA gene, cloned in pUC57 at BstBI and NotI site, was obtained commercially (Genscript, NJ, USA). The construct was excised out of the pUC57 vector and cloned in pPICZαB (Invitrogen, Thermo Fisher Scientific, USA) at BstBI and NotI sites downstream of alcohol oxidase (AOX)1 promoter as per standard protocols [27]. A two-copy construct was also prepared in a way that each expression cassette contained its own promoter sequence. The recombinant vector acted as a donor as well as an acceptor of HSA sequence. For this, the plasmid pPICZαB containing the CO-HSA gene was first digested with SmaI. After heat inactivation of the enzyme, the linearized DNA was run on 1% agarose gel and DNA extracted using a gel extraction kit (Qiagen GmbH, Hilden, Germany). Half of the DNA was digested with BglII and the other half with BamHI separately. After heat inactivation of the enzymes, the reaction products were run on 1% agarose gel and the longer fragments (harboring the AOX1 promoter, HSA gene, and the terminal region) were excised and DNA isolated by gel extraction kit. These two fragments were ligated in a reaction volume of 15 µL and 1U T4 DNA ligase (NEB, USA) was added. The scheme for construction of the two-copy construct is shown in Figure 1. Incubation was carried out at room temperature for 30 min followed by overnight ligation at 4 °C. The single and the two-copy plasmid constructs were transformed into *E. coli* DH5α for sequence confirmation and for large-scale preparation of the vector. For transformation into *P. pastoris*, the single- and two-copy vectors were first linearized with SmaI and then electroporated into competent *P. pastoris* cells. Zeocin was added at 100 mg/L (for selection of single-copy construct) and 500 mg/L for selection of two-copy transformants. The copy number was confirmed by Southern blotting in the finally selected transformants (data not shown).

### 2.3. Animal Cell Lines and Cell Proliferation Assay Kit

Vero cell line (Kidney epithelial cells derived from African Green monkey) was obtained from ICGEB, New Delhi. d-MEM medium, trypsin, FBS (Fetal Bovine Serum) were obtained from Gibco, Thermo Fisher Scientific, USAThe cells were propagated on d-MEM medium at 37 °C, 5% CO_2_*/*humified air, as described [28]. The MTT (3-[4,5-dimethylthiazol-2-yl]-2,5 diphenyl tetrazolium bromide) assay kit was procured from HiMedia Labs, Delhi, India.

### 2.4. Cultivation of Recombinant HSA Producing P. pastoris on Unoptimized and Optimized Medium and Optimization of HSA Production through Response Surface Methodology

The single and the two-copy containing recombinants of *P. pastoris* transformants were screened for production of HSA in 20 mL BMMY medium in 100 mL shake flask followed by cultivation of the selected clones in 100 mL as specified (Easy Select Pichia Expression kit, Invitrogen, USA). The cultures were harvested at the end of 144 h for analysis of HSA (by running on SDS-PAGE). Cell growth, total extracellular protein, and HSA production were monitored every 24 h until the end of 144 h.

The optimized medium was developed by optimization of three variables selected through Plackett-Burman methodology for which DOE software (JMP v10.0, SAS, North Carolina, USA) was used. The initial screening involved seven independent parameters (temperature, initial cell density, pH, aeration, initial methanol, sorbitol, and peptone concentration in the production medium) with four unassigned variables (referred to as dummy variables D1–D4). Each variable was examined at a high (+1) and low (−1) level, the details of which are provided in Table S1 of the Supplementary file. This set of 12 experiments was performed in duplicate to evaluate the effect of each parameter on extracellular HSA, as monitored by gel densitometry on SDS-PAGE. This design is based on the first order polynomial as shown below:

Y = β_0_ + ∑ β_i_X_i_ (1)

where Y is the response and refers to HSA produced (in mg/L), β_0_ is the coefficient of the model and β_i_ is the linear coefficient, and X_i_ refers to independent factor level. Based on the results obtained with the Plackett-Burman design, three factors, namely temperature, methanol, and peptone concentration, were selected for statistical optimization by face-centered central composite design (CCD) (software, JMP v10.0) as detailed in Appendix A. Each factor was used at three levels (−1, 0, +1) and six additional runs were also planned at center point resulting in a total of 20 experiments (for details, see Table S2 in the Supplementary file). The second-order polynomial was then used to identify the relationship between the factors and HSA production. The following quadratic regression model was used, in which the linear terms, the square terms, and the interaction terms are incorporated and response Y is predicted:

Y = β_0_ + ∑ β_i_X_i_ + ∑ β_ii_X_i_^2^ + ∑ β_ij_X_i_X_j_ (2)

_i_     _ii_  _ij_

where β_0_ is the regression coefficient, β_i_ is the linear coefficient, β_ii_ is the quadratic coefficient, and β_ij_ is the interaction coefficient between parameter i and j. X_i_ and X_j_ are the independent factors. JMPv 10.0 software was used for designing the experiments and also to draw 3D surface response. The response was evaluated in terms of production of stable HSA (as measured on SDS-PAGE by gel densitometry) by culturing single-copy (Clone # 52) and two-copy constructs (Clone #s 10 and 14) and comparing the data with that obtained on standard Invitrogen (unoptimized) medium. Biological and cell proliferation assays (see Analytical methods) were carried out with the purified HSA.

### 2.5. Purification and Cell Proliferation Assay of Recombinant HSA

The culture supernatant (90 mL), obtained from 144 h grown culture of Clone # 14, on optimized medium, was ultra-filtered using a stirred Amicon cell (Merck-Millipore, Sigma-Aldrich, USA) witha 10 kDa membrane. The retentate was subjected to size-exclusion chromatography using Toyopearl HW-50F resin (hydroxylated methacrylic polymer with a pore size of 12.5 nm, Sigma-Aldrich, USA) with a packed bed volume of 25 mL using FPLC ((Pharmacia, Sweden). Elution was performed with 0.1 M phosphate buffer, pH 6.0, at a flow rate of 1 mL/min. Fractions of 1 mL were collected and analyzed for total protein and HSA concentration. Fractions containing electrophoretically pure HSA were pooled and used for cell proliferation assay.

### 2.6. Transcriptome Analysis

Transcriptome analysis of two biological replicates of recombinant Clone # 14, cultivated on optimized and unoptimized medium, was carried out. The experimental flow chart is shown in Figure S1 in the Supplementary file. Total RNA was prepared from cells as described in Appendix B. The RNA prep was checked for quality by gel electrophoresis and then outsourced (Eurofins Genomics Pvt. Ltd., Bangalore) for preparation of paired end (PE) library (Illumina). The assembled transcripts were obtained and the remaining work was carried out in-house where the transcripts were clustered into Unigenes and coding sequences (CDS) covering 90% sequence coverage. Unigenes were quantified using RNA-Seq by Expectation Maximization (RSEM) package. Sample-wise Unigenes were identified based on Fragment per kilo base of transcripts per million reads (FPKM) estimated by RSEM (FPKM ≥ 1). The detailed work flow is explained in Appendix C. The predicted CDS were mapped to KEGG ortholog database *Komagataella phaffii* followed by classification under five categories: Metabolism, genetic information processing, environmental informational processing and cellular processes. A complete hierarchical analysis was performed on 100 differentially expressed Unigenes identified in experimental (optimized medium) and control (unoptimized medium) conditions. The data were analyzed by hierarchical clustering through heat map and Volcanic plot. Differentially expressed transcripts were plotted using Venn diagram software (http://bioinformatics.psb.ugent.be/webtools/Venn/), and DESeq (https://omictools.com/deseq-tool).

### 2.7. qPCR Validation of the Identified Target Genes

Quantitative polymerase chain reaction (qPCR) was carried out of four upregulated and three downregulated genes for confirmation of some of the target genes. The primers used for the purpose are shown in Table 1. For this, total RNA was purified from Clone # 14 grown for 120 h. A thick slurry of cells was poured through a syringe into liquid nitrogen to collect beads of ~4 mm diameter. The details of cDNA preparation and qPCR reactions are given in Appendix B. Glyceraldehyde 3-phosphate dehydrogenase (GAPDH) was used as housekeeping-reference gene with X-33 as the control host. The fold difference in the transcript levels was calculated as follows [29]:

ΔCt target = Ct _target gene_ − Ct _reference_; Fold expression = 2 ^−ΔCt^ (3)

ΔCt target = Ct _HSA gene_ − Ct _GAPDH_ (4)

### 2.8. Analytical Methods

Cell growth was monitored by measuring O.D._600_. Total protein was estimated by Bradford method. Extracellular protein profile and HSA production was monitored by loading 20 µL of cell-free supernatant on 12% SDS-PAGE. Staining and de-staining were carried out using standard methods. HSA was quantified by gel densitometry (Gel doc Analyzer, Bio-Rad, USA) as described previously for the granulocyte colony-stimulating factor [30]. Trypsin digestion and matrix-assisted laser desorption/ionization-time-of-flight (MALDI-TOF) analysis of the excised HSA band from the gel was carried out as per standard protocols [31].

Sandwich ELISA was used for measuring HSA in the culture supernatant using goat anti-human albumin as the primary antibody and horse radish peroxidase conjugated goat anti-human albumin as the secondary antibody. Standard HSA, provided in the kit (Bethyl laboratories, USA), was used for quantitation of the response. The details of the protocol are described in Appendix D. MTT assay was carried out according to the American Type Culture Collection (ATCC) [28]. Adherent cells were released by surface trypsinization. Approximately 5000 cells, in 100 µL of D-MEM medium, were placed per well along with the control (blank) in a 96-well plate. The cells were incubated for 6–48 h at 37 °C under 5% CO_2_/humidified air with purified HSA. MTT reagent (10 µL) was added to each well and cells incubated for 2–4 h and formation of intracellular purple precipitate monitored. After complete precipitation, 100 µL of detergent was added to stop the reaction. Supplementation with 10% FBS or 5% FBS (serving as positive controls), 0% FBS (negative control), 1 g/L purified HSA + 5% FBS, 0.5 g/L purified HSA + 5% FBS, and commercial HSA + 5% FBS was carried out in separate experiments. The assay was monitored for seven consecutive days in three technical replicates for each sample and O.D. was taken at 595 nm, considering day 0 as the control.

## 3. Results

### 3.1. Codon Optimization of Native HSA and Production in P. pastoris

**Figure 2:**
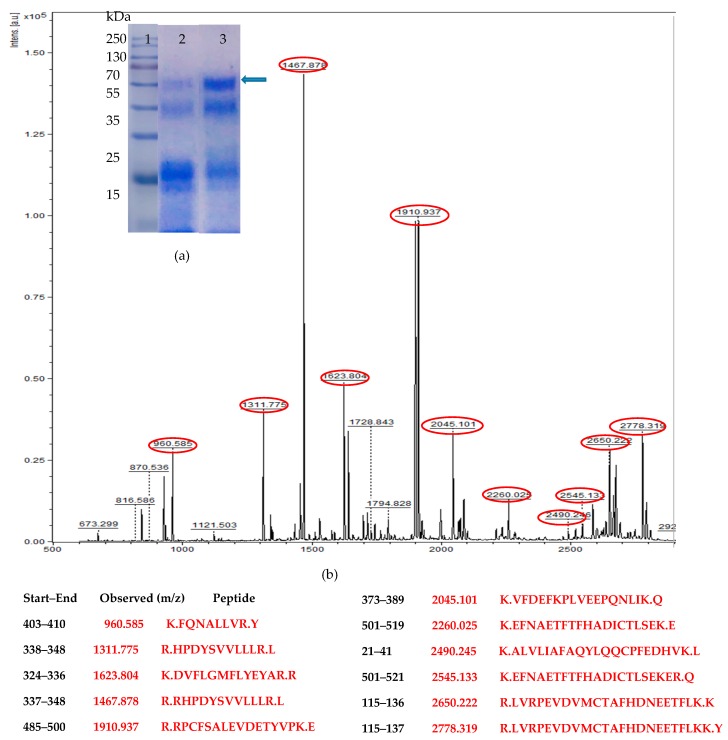
(**a**) Sodium dodecyl sulfate-polyacrylamide gel electrophoresis (SDS-PAGE) analysis of the total extracellular protein produced under unoptimized (Standard Invitrogen) conditions, Lane 1: Protein Ladder. Culture supernatant (20 µL) from Clone # 52 (single copy HSA): Lane 2, and Clone # 14 (two-copy HSA): Lane 3. Arrow indicates the HSA band. (**b**) Matrix-assisted laser desorption/ionization-time-of-flight (MALDI/TOF) peptide spectrum of the protein band from Clone # 14.

The human HSA gene codes for a protein of 609 amino acids including signal sequences and the mature form of the protein has 585 amino acids. These were optimized based on codon usage frequency, listed in the Kazusa database [26]. The final list of triplets is shown in Supplementary Table S3. The Codon Adaptation Index (CAI) was 0.74, slightly less than the ideal range of 0.8–1.0 [32]. A clear bias was seen for several triplets such as AGA (for Arg), GAT (for Asp), TGT (for Cys), TTG (for Phe), CCA (for Pro), TCT (for Ser), ACT (for Thr), and GTT (for Val). The extracellular production of HSA was confirmed by monitoring the extracellular protein on the SDS-PAGE gel. In order to select a `good’ producer strain out of a total of 48 positive (based on PCR analysis with HSA primers) transformants obtained with single-copy cassette, 15 were screened in 20 mL BMMY medium and an average extracellular protein of 120 ± 20 mg/L obtained (data not shown). Cl # 52 was selected based on production of high extracellular protein (~230 ± 35 mg/L) at 100 mL level. Trypsin digestion of the band at ~66 kDa position (Figure 2a) followed by MALDI-TOF analysis indicated presence of several peptide fragments (Figure 2b). These could be matched to theoretical peptides expected after trypsin digestion of intact HSA. This confirmed that the protein resolved at ~66 kDa position in the sodium dodecyl sulfate-polyacrylamide gel electrophoresis (SDS-PAGE) was that of HSA.

A preliminary investigation of role of aeration indicated lower aeration (by way of cultivation in non-baffled flasks) to favor high extracellular protein production (data not shown) and was thus used in subsequent experiments. Similarly, for the two-copy constructs, 31 transformants were initially screened (See Figure S2 in the Supplementary file), out of which Cl #s 10 and 14 consistently produced high (660 ± 15 and 590 ± 10 mg/L, respectively) levels of extracellular protein. However, the extracellular HSA was found to undergo degradation after 120 h of fermentation (when monitored on SDS-PAGE) for both the single and the two-copy constructs (Figure 2a).

### 3.2. Medium Design and Evaluation of Secreted HSA

**Figure 3:**
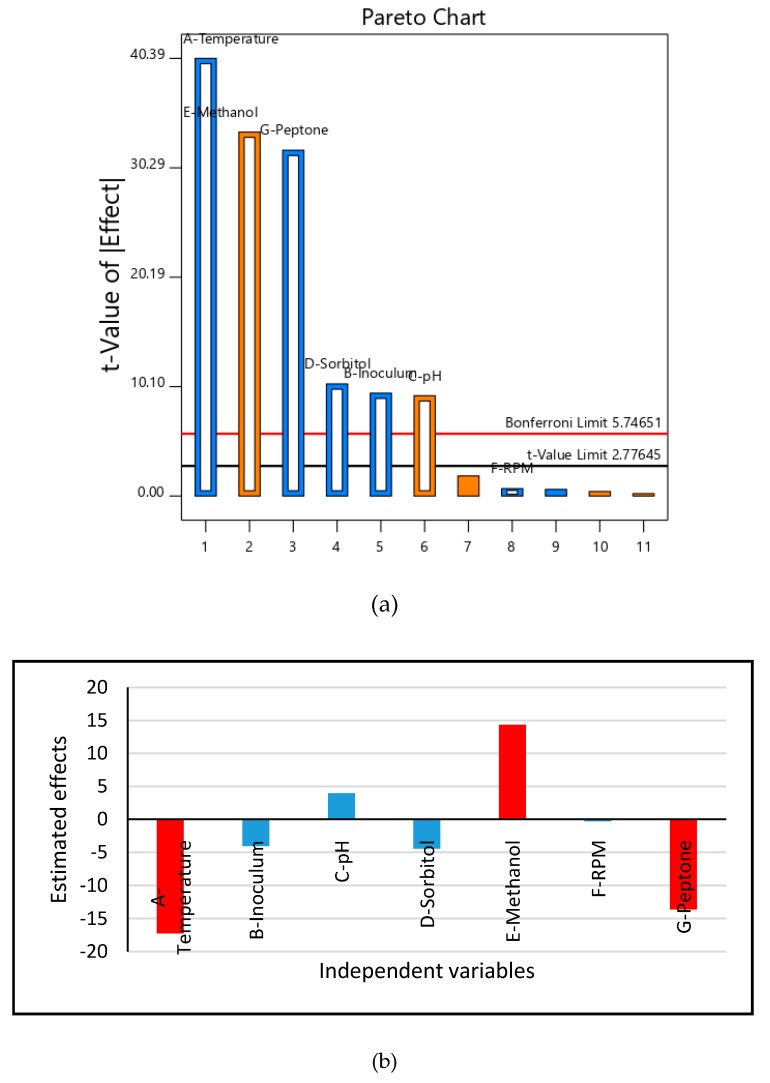
(**a**) Pareto chart obtained from the Plackett-Burman design representing the order and % contribution of each parameter on production of stable HSA. Blue and orange color represent the negative and positive contribution respectively. (**b**) Estimated effects of independent variables on HSA production by Clone # 14. Red indicate the most significant while blue indicate nonsignificant parameters.

In order to optimize cultivations conditions under which stable production of HSA could be obtained, a number of factors reported to affect extracellular protein production in the *P. pastoris* system were investigated. In the first stage of screening, the Plackett-Burman design was used to assess the effect of seven factors (Appendix A and Table S1 in the Supplementary file), namely, temperature, inoculum level, pH, sorbitol concentration, methanol level, aeration as measured by shaking at different revolutions per minute (RPM) and peptone concentration on HSA production, and stability in the culture medium. The result of the experiment conducted with Clone # 14 indicated variability in HSA production, and as judged on the gel, it varied from ~240 (Run # 4) to ~290 mg/L (Run # 12) (Table S1 in the Supplementary file). The use of multiple-regression model allowed a correlation to be made between the seven factors and production of stable HSA. From the results of the Pareto chart (Figure 3a) and estimated effects (Figure 3b), it was observed that temperature, methanol level, and peptone concentration in the induction phase had the maximum effect on the production of stable HSA. Their percentage contribution was 40, ~28, and 25%, respectively, amounting to 93% of the effect. Analysis based on analysis of variance (ANOVA, see Table S4 in Supplementary file) indicated that the *p* value of the model was 0.0022 (< 0.05). Hence, the model parameters were significant. The significant variables were identified and are shown in Table S4. The coefficient of determination value (R^2^) was 0.9990 which showed that 99.9% of the variation in the HSA production value could be demonstrated by the independent factors chosen in this study. An adequate precision value of 82.18 (which was >> 4) is considered desirable and indicated that the model could be used for exploring the design space.

Based on the findings of the Plackett-Burman design, temperature, methanol level, and peptone concentration were chosen as parameters in the face centered-CCD experiments and the results are shown in Table S2. As seen, the combination of the three parameters which extended extracellular stable HSA production from 290 to 350 mg/L were identified. A good correlation was observed between the predicted and the actual values of HSA obtained through the experiments. Regression analysis of the data indicated that the coefficient of determination (R^2^) was 0.994, which indicated that 99.4% of the variation could be explained by the model parameters. The adjusted R^2^ was 0.9886, which confirmed the model to be significant. The ANOVA values of the regression model are shown in Table S5 of the Supplementary file. The *p* values, which indicate significance of the coefficients and also allow us to understand the interaction between the chosen variables, were calculated. Examining the probability values of the coefficients indicated that all three parameters, i.e., temperature, methanol, and peptone concentration, interaction between temperature and peptone, and quadratic effects of the three studied parameters demonstrated maximum effect on HSA stability as *p*-values were less than 0.05. Importantly, the interaction between temperature and peptone concentration was found to be most significant, followed by the interaction between methanol and peptone. A good fit of the model was predicted and a *p*-value < 0.0001 indicated that there was less than 0.01% chance that the F-value of 183.74 could have occurred due to noise. To determine the relationship between HSA production and the parameters (temperature, methanol, and peptone concentration) and also to arrive at optimal concentration of each parameter, the second-order polynomial was obtained. This defines the predicted output (production of stable HSA) in terms of the independent parameters:

Y = 344.9 − 11.6X_1_ − 8.1X_2_ − 25.6X_3_ + 1.4X_1_X_2_ + 10.1X_1_X_3_ + 5.1X_2_X_3_ − 22.8X_1_^2^ − 33X_2_^2^ − 28.4X_3_^2^ (5)

where Y is the predicted concentration of HSA, and X_1_, X_2_, and X_3_ are temperature, methanol level, and peptone concentration, respectively.

**Figure 4:**
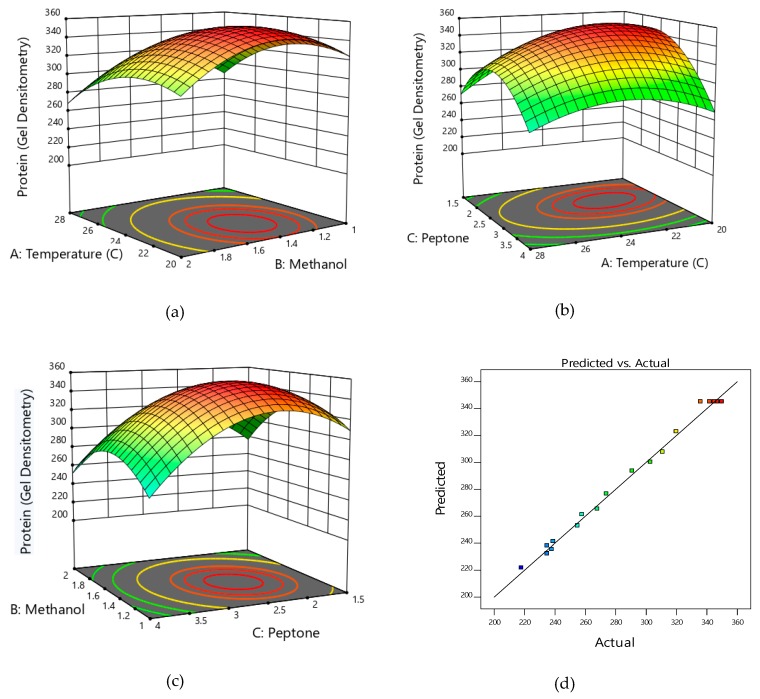
Three-dimensional response surface graphs and contour plots indicating the effect of individual parameters and the interaction between two parameters. Effect of combined effect of temperature and methanol (**a**), peptone and temperature (**b**), and methanol and peptone on HSA production (**c**). Correlation between the predicted and the observed response (Y as HSA produced in mg/L) (**d**).

**Figure 5:**
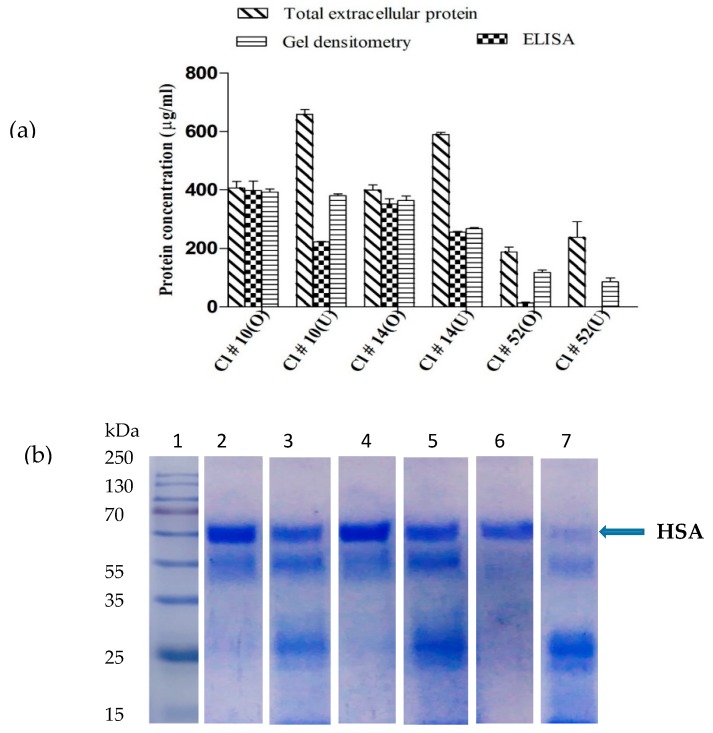
(**a**) Total extracellular protein production, ELISA, gel densitometry analysis of the secreted HSA in two-copy Clone #s 10, 14, and in single-copy Clone # 52 under optimized (O) and unoptimized (U) media conditions. (**b**) SDS-PAGE analysis of the culture filtrate from clones mentioned in (**a**). Lane1- Protein Ladder, Lanes 2, 4- Clone #s 10, 14, under optimized conditions and Lanes 3, 5 on unoptimized medium. Lanes 6, 7 show the extracellular protein profile of Clone # 52 under optimized and unoptimized condition, respectively.

The interrelationship between any two parameters was predicted by the 3D contour profiler through response surface graphs in orthogonal projection (Figure 4a–c). As seen in Figure 4a, as temperature increased from 20 to 23 °C, HSA production increased. However, beyond 24 °C, HSA production decreased. Also, an increase in methanol level from 1% to 1.4% resulted in an increase in HSA production, while any further increase lead to decrease in obtaining stable HSA. Figure 4b,c describes the interdependence between temperature and peptone and between peptone and methanol, respectively. Temperature between 22 to 24 °C and peptone between 2.2% to 2.6% would evoke the maximum response. Figure 4c indicated that peptone between 2.2% to 2.7% and methanol between 1.3% to 1.6% yielded maximum production of stable HSA. A good correlation was observed between the predicted and the observed values (Figure 4d). The predicted model was validated by carrying out experiments at the optimized conditions and these were combination of 1.5% methanol, 22 °C temperature, and a peptone concentration of 2.5%. Under these optimized conditions, the one copy construct (Clone # 52) yielded a total extracellular protein level of 190 ± 15 mg/L (Clone # 52) and 400 ± 20 and 410 ± 10 mg/L (Clone #s 10 and 14). These were lower than those obtained on the unoptimized medium (Figure 5a), while the HSA produced on the optimized medium was stable at the end of 144 h of fermentation (Figure 5b). The level of HSA, as judged from gel densitometry for the two-copy constructs, equaled the total extracellular protein indicating little degradation of the synthesized protein. The quantity of HSA produced, based on ELISA, constituted about 95% of the total protein on the optimized medium compared to 45% on the unoptimized medium (Figure 5a). This was more prominently seen for the two-copy than for the single-copy construct.

**Figure 6:**
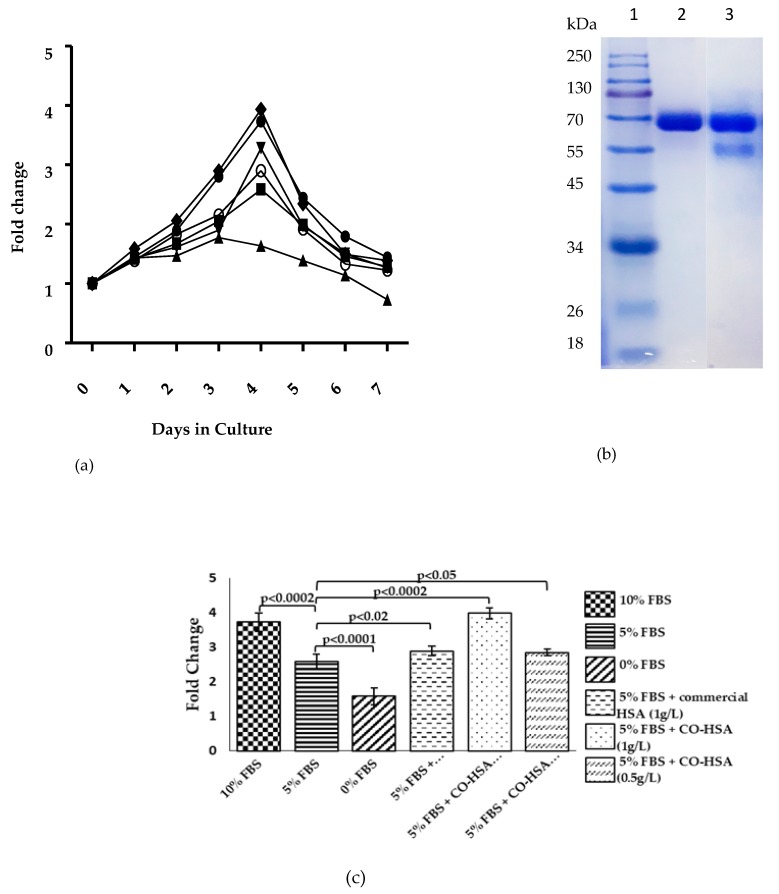
(**a**) Cell viability (MTT) assay with Vero cell lines. Fold change in proliferation was measured in the absence of any fetal bovine serum (FBS) (▲), 5% FBS (■) or 10% FBS (●), 5%FBS + 1 g/L commercial HSA (▼), 5% FBS + purified HSA 0.5 g/L (○), or 5% FBS + purified HSA 1g/L (◆). (**b**) SDS-PAGE analysis of purified HSA from Clone # 14 cultivated on optimized medium. Lane 1: Marker, Lane 2: Commercial HSA (4 µg), Lane 3: 4 µL fast protein liquid chromatography (FPLC) fraction. (**c**) The statistical significance of the difference was determined by paired *t*-test taking 5% FBS as standard reference for cell proliferation experiments.

The cell proliferation assay was carried out with purified HSA. For this, HSA was purified from the culture filtrate of Clone # 14, cultivated on the optimized medium. The purified HSA appeared as a major band around 66 kDa. A minor truncated form of HSA was detected at 55 kDa, the identity of which was confirmed by MALDI/TOF analysis. In the cell proliferation assay, 10% FBS supplemented medium provided an O.D. of 3.75 followed by 5% FBS, which allowed an O.D. of 2.5 on fourth day of incubation (Figure 6a). In the medium, where no FBS was added, the O.D. decreased and was followed by cell lysis due to deprivation of FBS. The addition of 1 g/L of purified HSA (Figure 6b) with 5% FBS resulted in cell proliferation response, which was superior than that obtained when commercial HSA was added with 5% FBS. It provided a response equal to that obtained with 10% FBS supplementation. Significant enhancement in proliferation was also observed when the purified HSA was added at 0.5 g/L (Figure 6c).

### 3.3. Transcriptome Profiling of Recombinant P. pastoris Cultivated on Optimized and Unoptimized Medium

An analysis of the transcriptome library indicated the fragment size distribution to be around 341 and 391 bp from cells cultivated on optimized (O) and unoptimized (U) medium. A search of predicted Unigene sequences in the National Center for Biotechnology Information (NCBI) nonredundant (Nr) protein database indicated the majority of the hits were against *Komagataella phaffii* GS115. The transcripts, based on gene ontology (GO) IDs, were assembled into cellular components (1021(U), 1102(O)), molecular functions (1519(U),1589(O)), and biological functions (1487(U), 1566(O)), where the transcript numbers were followed by medium of cultivation, U standing for the unoptimized and O standing for optimized medium (see Figure S3a,b in the Supplementary file). Categorization was also carried out based on CDS and the data are shown in Figure S4a,b in the Supplementary file. Differentially transcribed genes, with log_2_fold values from 0.5 to 4.5, were identified. A number of genes (between 6–15) were either up- or down-regulated (some by as much as four-fold) on the optimized medium and these are displayed in a volcanic plot (see Figure S5 in the Supplementary file). These CDS were mapped on the KEGG pathways and classified into the categories of metabolism, genetic information processing, environmental information processing, and cellular processes (see Table S6 in the Supplementary file). The details of the CDS of each category were provided (see Figure S6a in Supplementary file). Percentage-wise distribution of up- and down-regulated genes showed that genes related to methanol metabolism, translocation, glycosylation, protein folding, and proteasome were upregulated, whereas 30% of the genes involved in the endoplasmic reticulum associated degradation (ERAD) pathway were downregulated. A total of 2383 genes were re-evaluated and out of 983 unique genes, the Venn diagram displayed 975 genes expressed under both conditions (Figure S6b in Supplementary file), 16 that were upregulated, and 15 that were downregulated on optimized and unoptimized medium. Out of the 16 up- and 15 down-regulated genes, six and two exclusively belonged to each category and their functional details, based on mapping on KEGG pathways, were described (Supplementary Figure S6c,d).

**Figure 7:**
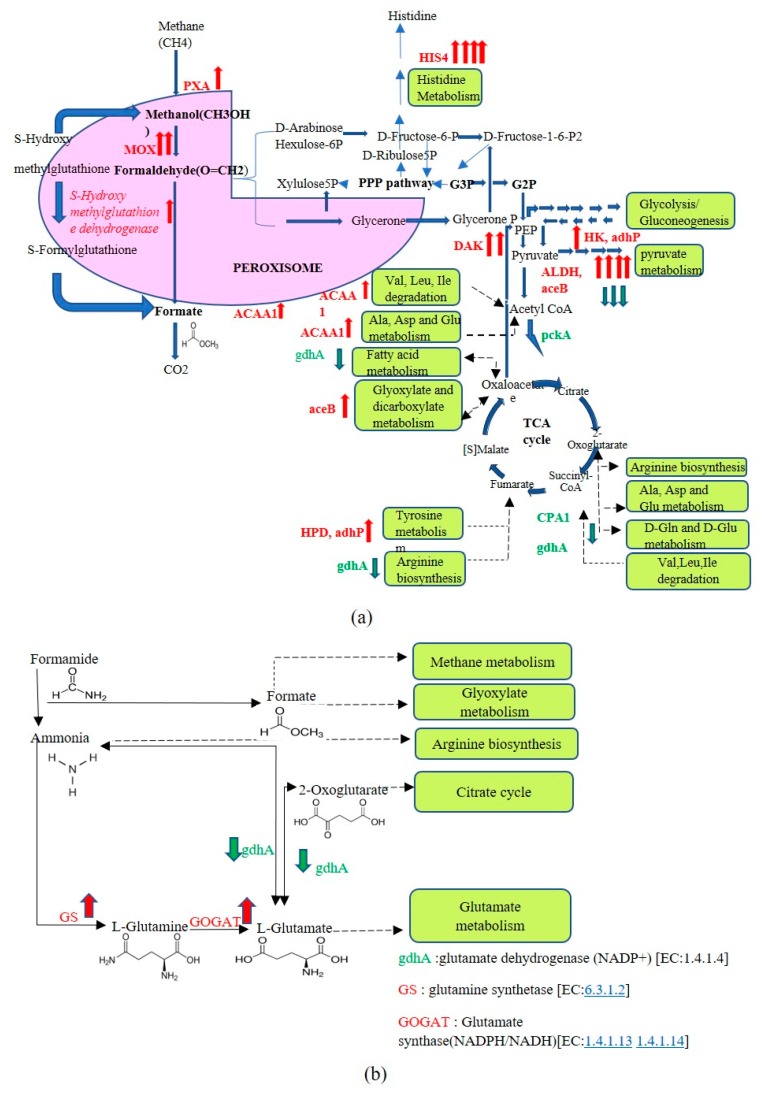
(**a**) Genes regulated in carbon metabolism under optimized conditions (**b**) Genes regulated in nitrogen metabolism according to KEGG pathways under optimized and unoptimized conditions. Intensity of up- (shown in red) and down-regulated (green) genes is shown by the number of arrows. One arrow represents log_2_fold unit change of 1.

The up- and down-regulated genes were assessed for their contribution to carbon and nitrogen metabolism, transcription, translation, folding, and secretion. Only those genes were described for which a clear functional role could be assigned. As seen in Figure 7, genes encoding for enzymes involved in methanol metabolism, such as alcohol oxidase (MOX), *S*-hydroxymethyl glutathione dehydrogenase (frmA), hexokinase (HK), dihydroxyacetone kinase (DAK2), were upregulated (by varying degrees) on the optimized medium. Upregulation was also observed for genes involved in catabolism of fatty acids (Acetyl-coA acyl transferase1 or ACAA1), pyruvate (alcohol dehydrogenase or ALDH malate synthase A or ACE B, propanol preferring alcohol dehydrogenase or ADH P), amino acids (ACAA1, 4 Hydroxyphenyl pyruvate dioxygenase, or HPD, histidinol dehydrogenase HIS4, ADH P), and peroxisome transport (peroxisomal long-chain fatty acid import protein or PXA) (Figure 7a). However, a few key enzymes of pyruvate metabolism like phosphoenol pyruvate carboxykinase (PCKA) and amino acid metabolism (glutamate dehydrogenase or GDH A, carbamoyl-phosphate synthase small subunit or CPA1) were found to be downregulated. Importantly, an alternate source to nitrogen supply (other than that in which GDH A operates) was observed to be operating under optimized conditions by way of upregulation of genes encoding Glutamate synthase (GOGAT)/Glutamine synthetase (GS) (Figure 7b).

**Figure 8:**
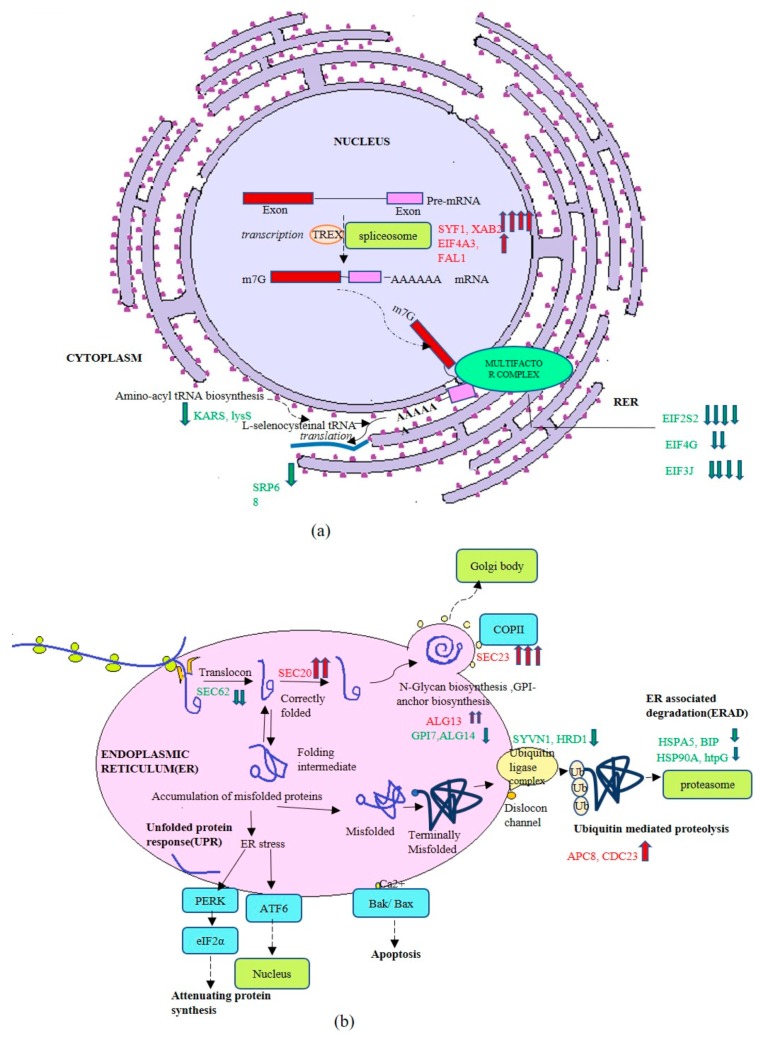
(**a**) Genes regulated in post-transcriptional pathway under optimized and unoptimized conditions. (**b**) Genes regulated in transport of protein to ER, folding and secretory pathway under optimized and unoptimized conditions.

Major differences were not observed in the transcription and translational machinery components on optimized and unoptimized medium, while some genes were upregulated by as much as four-fold (XPA-binding protein 2 or XAB2, gene coding for pre-mRNA-splicing factor, homolog of SYF protein) or one-fold (Translation initiation factor 4G or EIF4A3 and FAL1: ATP-dependent RNA helicase or FAL1). These three genes are related to RNA post-transcriptional events such as spliceosome functioning, providing splicing factors to active genes [33], and transcription-coupled DNA repair [34]. Genes encoding Translation initiation factor 3, subunit J (EIF3J), Translation initiation factor 2 subunit2 or EIF2S2, and Translation initiation factor 4G orEIF4G were downregulated. Moreover, lysS encoding, lysyl tRNA synthetase, KARS, and signal recognition particle SRP68 were observed to be downregulated. The data pointed toward increased transcriptional activity while slowing down translational processes (Figure 8a). An investigation of the up- and down-regulated genes mapped on folding and secretory pathway indicated that membrane transport proteins such as SEC20, SEC23, and *N*-glycan biosynthesizing enzyme ALG13:beta-1,4-*N*-acetylglucosaminyltransferase, ALG14 (coding for the UDP-GlcNAc transferase catalyzing a key step in endoplasmic reticulum *N*-linked glycosylation), were upregulated. Of interest is that protein involved in the cell division cycle (CDC23) and anaphase promoting complex (APC8) were upregulated. The gene encoding SEC62, encoding for protein translocation into endoplasmic reticulum, and genes coding for GPI anchor biosynthesis (GP17) and ALG14 were among the downregulated genes. Several genes encoding for proteins involved in ubiquitin mediated proteolysis, including chaperones, were downregulated, including ubiquitin protein ligase synoviolinor or SYVN1, HMG-CoA reductase degradation protein coded for by *HRD*1, and chaperones like HSP90, HSPA5, BIP, and htpG. All these are a part of endoplasmic-reticulum-associated protein degradation (ERAD) pathway which promotes degradation of misfolded proteins (Figure 8b). This indicated that protein mis-folding did not occur on the optimized medium.

**Figure 9:**
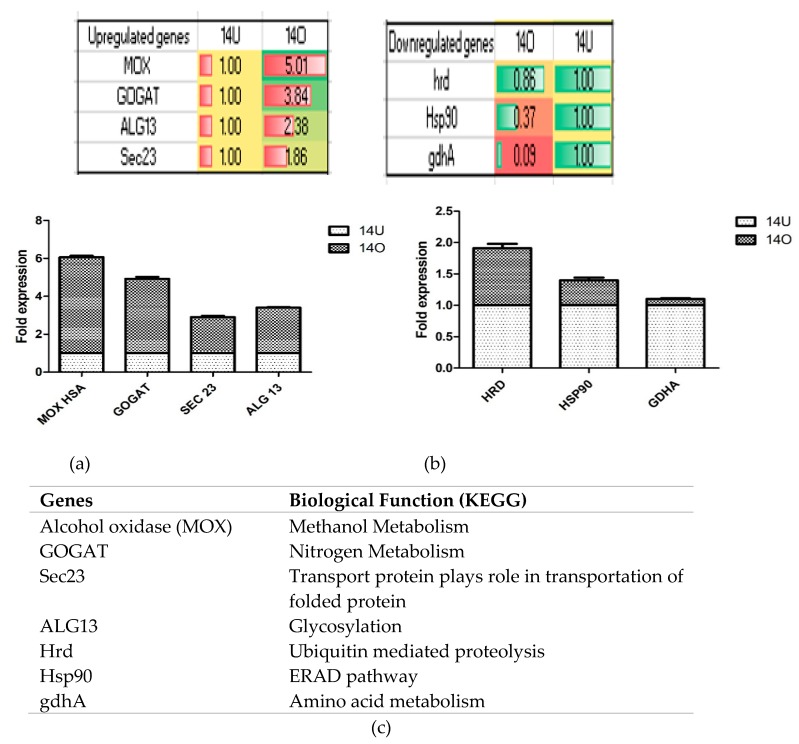
Qualitative polymerase chain reaction (QPCR) analysis of some (**a**) up- and (**b**) down- regulated genes under optimized vs. unoptimized media conditions. (**c**) Biological function of the genes mentioned in (**a**) and (**b**).

The up- and down-regulated transcription of a few genes was validated by qPCR and the results are shown in Figure 9. As seen, *MOX* showed the highest fold change of 5.01 ± 0.11 followed by *GOGAT* (3.84 ± 0.02-fold). *ALG13* and *SEC23* were upregulated by 2.38 ± 0.04, 1.86 ± 0.09-fold respectively relative to the control conditions (Figure 9a). Among the downregulated genes, *HRD*, *HSP90*, and *GDH*A showed a 0.86 ± 0.1, 0.37 ± 0.07, and 0.09 ± 0.02-fold change under optimized conditions (Figure 9b). The specific function of these genes is described in Figure 9c.

## 4. Discussion

*P. pastoris* remains a sought-after expression platform for production of a large number of proteins, including biopharmaceuticals [1,35], and, with the availability of a number of cultivation strategies [19,36], has yielded levels of extracellular protein in the range of 4–12 g/L. In addition, a number of genetic strategies are available with respect to overproduction of heterologous proteins [37]. Application of any one of these does not ensure stability of the secreted product. This issue is relevant in case the therapeutic is injected in large dose in the blood stream. HSA 20% formulation, for instance, is injected at a level of 0.5–2.0 mg/kg body weight (depending on the disease) as a blood volume manager [38].

In this study, we have reported, for the first time, the development of a medium, using statistically available tools, that supported stable production of HSA in rich medium at shake flask level. The stable production was achieved for one-copy as well as the two-copy containing CO-HSA constructs, indicating that physiological changes occurred when these were cultivated on the optimized medium. The production of HSA was found to be affected by external medium components and the operating conditions such as temperature, pH, and aeration to varying levels. For initial screening of the factors, a two-level Plackett-Burman statistical design was used and seven factors were evaluated during the methanol induction phase, namely temperature, inoculum level, pH, sorbitol concentration, methanol level, aeration as controlled by RPM, and peptone concentration. Plackett-Burman is a quick and reliable method for screening a large number of parameters and leads to identification of significant factors. This method was successfully used to identify three major factors which together could account for 93% of the influence on stable production of HSA. Based on the ANOVA results, the model was considered to be adequate to further arrive at optimized values of temperature, methanol and peptone concentration.

Temperature of cultivation during the methanol induction phase has been widely reported to affect foreign protein production in *P. pastoris* with productivity decreasing with an increase in temperature. Lowering of temperature in the production phase has been reported to result in increased extracellular protein production with decreased cell lysis and protease activity, and the effect has been linked to lowered specific growth rates [39,40]. This was also attributed to better protein folding rates at 23 °C [37], facilitating secretion and suppressing the ERAD pathway. It has been reported [7] that recombinant human interleukin-10 (rhiL-10) expressed in *P. pastoris* under *AOX1* promoter produced lesser amount of protein when cultured at 30 °C compared to 20 °C. It was observed that accumulation of the protein in the ER lead to ER stress triggering the unfolded protein response (UPR) pathway. Similar effect of lowered temperature has been demonstrated earlier on production of recombinant LIP1 protein of *Candida rugosa* [41].

The amount of methanol supplied to cells is extremely important as it serves to induce the *AOX1* promoter and is also used as a carbon source during the methanol induction phase in the BMMY medium. Too little methanol leads to poor induction and growth, while a high concentration leads to the accumulation of formaldehyde (the first product of methanol metabolism) which leads to cellular toxicity [16,42,43]. Methanol consumption is an exergonic process leading to heat generation. Thus, *P. pastoris* fermentations need to be temperature-controlled (Easy Select *Pichia* Expression kit, Invitrogen, USA). It has been suggested to supply a second carbon source such as glycerol, lactose, or sorbitol [5,19,44], which can effectively serve as a carbon source while low concentration of methanol can bring about induction. The results of primary screening confirmed that methanol levels impacted the HSA production in a very significant way.

Initial screening also identified peptone levels to significantly affect HSA production. Peptone is reported to influence protease production which are responsible for degradation of extracellular proteins. In general, two major strategies have been reported for arresting this proteolytic degradation. One is based on the use of protease deficient strains such as SMD 1163(Δ*his*4Δ*pep*4 Δ*prb*1), SMD1165 (Δ*his*4 Δ*prb*1), and SMD1168 (Δ*his*4 Δ*pep*4) [45]. The drawback with these is that these grow slowly and thus affect cell biomass accumulation and consequently heterologous protein production. Lowered levels of peptone have been recommended for production of bikunin fused with HSA [46]. A rich yeast-peptone medium has been reported to suppress the utilization of methanol during lipase production from *Geotrichum candidum* expressed in *P. pastoris* [47].

In order to develop an optimized medium and identify most suitable temperature, a face-centered CCD strategy was used in which the response (Y) was measured in terms of production of stable HSA, rather than the total extracellular protein. This allowed strict control over an optimization process and allowed us to identify conditions under which the HSA produced constituted over 90% of the total cellular protein. The response surface curve also revealed a strong interaction between temperature and peptone, hitherto unidentified in the *P. pastoris* system. An increase in temperature has been correlated with an increase in cell lysis leading to increase in extracellular protease production [48]. Low concentration of peptone can also induce proteases. Hence, an adequate amount is required to support growth and arrest production of proteases. It is also important to note that the model-predicted optimum methanol concentration of 1.5% was supplied in two equal aliquots (initial 0.75%, followed by addition of 0.75% after 12 h period) which led to cell stability and better growth (see below). The HSA produced was biologically stable, and, it was concluded that it could substitute for the externally added FBS for proliferation of the Vero cell lines. Addition of a low concentration (0.1%) of purified HSA to 5% FBS supplemented culture resulted in cell proliferation that was equivalent to that achieved with supplementation with 10% FBS and significantly higher than that achieved with supplementation with commercially available HSA (Reliance Life Sciences, India) at same protein loading.

In order to identify the underlying physiological conditions affected in the optimized medium, transcriptome analysis was carried out. Instead of listing the genes, the focus was to assess the impact on cellular physiology. With respect to carbon metabolism, several enzymes of methanol metabolic pathway, such as, MOX and DAK2, were upregulated, indicating efficient methanol utilization. These are directly involved in conversion of methanol to fructose-1,6-bisphosphate, an important metabolite required for carbon assimilation. Along with this was identification of ALDH, which along with formaldehyde reductase is one of the three major alcohol dehydrogenases and is a part of the futile cycle which regulates the cellular content of formaldehyde and NADH in *Pichia methanolica* [49]. The upregulation of these genes in *P. pastoris* on optimized medium suggested presence of futile pathways that lower accumulation of formaldehyde. Such physiological condition also lowers cell lysis, which would explain lowered extracellular levels of proteins under optimized conditions. Other essential findings were higher metabolic activity (flux) of the TCA cycle in keeping with energy requirements (NADH, ATP) of actively growing cells, as reported [50,51]. Diversion of 2-oxoglutarate to d-glutamine and d-glutamate was found to be reduced by lowering the levels of GDHA, thereby increasing flux of metabolites in the TCA cycle. A similar flux in TCA cycle with upregulation of genes involved in methanol utilization pathway was also documented when differential gene expression was studied under methanol feed in *P. pastoris* when hybridized with probes of *S. cerevisiae* [52]. Several genes involved in biosynthesis of nucleotides and metabolism of cofactor (such as Pantothenate and CoA) were also upregulated, indicating that growth was facilitated under these conditions. Such a condition is also likely to de-stress the peroxisomes, which are otherwise impermeable to dinucleotides (NADP, FAD) and Acetyl CoA and depend on shuttles to maintain the cofactor pool [53]. Upregulation of some of these genes has been reported in protein processing and export pathways, peroxisome biogenesis when *P. pastoris* was shifted from glycerol to methanol medium [54], and also with *Hansenula polymorpha* [55]. A proteomic approach followed during methanol-induction phase also confirmed the upregulation of several of these genes during insulin production [56]. A strong impact was also observed on nitrogen metabolism and increased availability of nitrogen was accomplished by the cells by the upregulation of genes encoding GOGAT and GS. These enzymes are responsible for conversion of ammonia to l-Glutamate and can support glutamate availability for incorporation into various amino acids [57] and eventually protein synthesis. An increase in arginine metabolism was also observed and may help in nitrogen cycling [58].

Analysis of the transcript levels of genes associated with transcription, translation, and secretion indicated several components of translational machinery (EIF4G, EIF3J) to be downregulated, suggesting post-transcriptional buffering to avoid stress on folding and misfolding associated response by the cell. Such buffering has also been observed in *S. cerevisiae* [59]. In another approach, transcriptomic profiling carried out under simulated gravity compared to normal gravity conditions indicated that recombinant protein production in *P. pastoris* was correlated with the upregulation of genes involved in methanol utilization pathway, RNA polymerase synthesis, chaperone, protein transportation, and secretion [60]. The suppression of UPR pathways was confirmed from the observation that transcription of *HRD*1, gene encoding SVYN1, *Sel1*L, and genes encoding several chaperones (HSP90, HSPA5, BIP, HTPG) were downregulated. HRD1 protein is a principal ER-resident E3 ligase (along with SYVN1) that forms a complex with the ER-resident Sel1L (also known as mammalian Hrd3) and is responsible for degradation of a subset of misfolded proteins in the ER [61,62,63]. Many of these functions have also been correlated with lowered cultivation temperature in *P. pastoris* [7,42,64]. Moreover, no global regulation was found for vacuolar stress or the ERAD pathway genes. A fine tuning was thus observed between translational machinery and secretion of the recombinant protein. A comparative analysis of the differentially expressed genes identified in this study was carried out with differentially regulated genes under lowered methanol metabolism [54] and temperature [65] and the results are shown in Table S7 in Supplementary file. The up- and downregulation of several genes was found to be identical to that reported earlier [54,65], while some were found to be unique and are being identified for the first time (see highlighted genes in Table S7 in Supplementary file). Those that were upregulated pertained to methanol and nitrogen metabolism, energy generation, and transport proteins. Several were also downregulated and pertained to lowering of translation rates, chaperone availability, and proteasomal complex formation. These can serve as targets for strain engineering.

## 5. Conclusions

Experiments based on statistical design were chosen to identify factors affecting extracellular production of stable HSA in the culture supernatant of *P. pastoris*. Temperature, methanol level, and peptone concentration were found to affect HSA and optimization of these lead to development of an optimized medium. Next-generation sequencing techniques were used to obtain transcriptome data of a two-copy HSA construct cultivated on optimized and unoptimized (standard Invitrogen) medium. In total, accurate identification of 7665 CDS was made on the optimized medium and 7571 CDS on unoptimized medium. An analysis of 16 upregulated and 15 downregulated genes under two cultivation conditions was performed and the genes were mapped to pathways belonging to four categories. These were methanol, nitrogen metabolism, genetic information processing (including transcription and translation), folding, and secretion. Based on their up- or down-regulation, an attempt was made to understand the contribution of these genes to cell physiology and foreign protein production. Stable HSA production was associated with increased methanol metabolism, increased availability of nitrogen, increased cell growth, and decreased cell lysis. Proteolysis was avoided by controlling the translational rate of the proteins, which allowed proper folding and suppression of the UPR and ERAD pathways. Transcription of some of the key differentially regulated genes was also confirmed by qPCR studies.

## 6. Patents

Indian Patent Application no. 201711034803 (filed)

## Figures and Tables

**Table 1:** List of primers used in studying transcript levels of some up- and down-regulated genes under optimized/unoptimized conditions.

Primers	Sequence of Primers
FP Hsp90	5′CTGAGGAAGATGACAAGAAGCC3′
RP Hsp90	5′GAAGTGCTTAACAGCCAATGGG3′
FP gdhA	5′TTTCTCCGACTCTCCAGTCAAGTAC3′
RP gdhA	5′AGCCTCCAAAGTAGAACCCATG3′
FP HRD	5′GAACAGTCATCATCCCGAGAG3′
RP HRD	5′CTTTGTCCATACGAGAGAGATC3′
FP MOX	5′CAGAGGTTCCGCTTCTGATTAC3′
RP MOX	5′TTGGTACTCAGAAGCCCTCAAG3′
FP ALG13	5′GCTGATTCGAACACATAGAAGG3′
RP ALG13	5′GTCGGGTTTTATATCCCTGTG3′
FP Sec23	5′AACAATACGCTTTTGCTTGCTCC3′
RP Sec23	5′ATCACATTGGCCTTTCTCTTGG3′
FP GOGAT	5′GCTGCTATGGGAG CTGATGAA3′
RP GOGAT	5′GCAACGGCCAAG AATCATGTA3′

## Supplementary Materials

The following are available online at https://www.mdpi.com/2218-273X/9/10/568/s1, Figure S1: Bioinformatics workflow. Figure S2: SDS-PAGE analysis of culture filtrate produced by two-copy constructs. (a) Lanes 1–6: Clone # s are shown above the lane numbers. Lane 7: One-copy construct, Lane 8: Commercial HSA producing clone (com), Lane 9: Standard HSA (Commercial), Lane 10: Molecular weight ladder. (b) Lanes 1–6: Clone # s are shown above the lane numbers. Lane 7: One-copy construct, Lane 8: Commercial HSA producing clone (com), Lane 9: Molecular weight ladder, Lane 10: Standard HSA (Commercial). (c) Lanes 1–7: Clone # s are shown above the lane numbers. Lane 8: One-copy construct, Lane 9: Commercial HSA producing clone (com), Lane 10: standard HSA (Commercial). Figure S3: (a) Classification of genes based on Gene Ontology, b) WEGO plot showing GO for Unigenes. Figure S4: (a) Classification of CDS transcripts based on gene ontology IDs, (b) WEGO plot showing GO for CDS sequences. Figure S5: Volcanic plot representing differentially expressed genes. Figure S6: Percent distribution of up- and down-regulated genes. Table S1: Design of Experiments (DOE) using Plackett-Burman methodology (unit for temperature is degrees Celsius, inoculum is O.D. units, where 1 O.D._600_ unit corresponds to approximately 10^7^cells/mL, Sorbitol and peptone are in percentage (*w*/*v*), methanol in percentage (*v*/*v*). Table S2: Design of experiments (DOE) using Central Composite Design methodology (CCD). Table S3: Codon usage table of cloned HSA. Table S4: Regression statistics and ANOVA for Plackett-Burman design. Table S5: Analysis of variance (ANOVA) for quadratic model obtained from face centered CCD. Table S6: KEGG pathway classification of genes transcribed under optimized and unoptimized conditions. Table S7: A comparison of the up- and down-regulated genes obtained from studies involving methanol metabolism [54], temperature [65] and present study.

## Appendix A

Medium optimization through Plackett-Burman (PB) and Central Composite Design (CCD)

A lower and an upper limit was set individually for all the seven factors as shown in Tables S1 and S2 in the Supplementary file. Using these, a set of 12 experiments was statistically designed and carried out in triplicates using recombinant Clone # 14 containing a two-copy CO-HSA construct. Induction of HSA was carried out by addition of 1% methanol every 24 h, whereas the induction time was repeated every 12 h for 1.5% methanol (750 µL in each pulse) in order to avoid cell toxicity and cell death. Samples were removed every 24 h for monitoring cell O.D., pH, extracellular protein, and HSA in the culture filtrate. Both extracellular protein level and HSA stability were the responses studied. The experiments were carried out for 144 h post-methanol induction. Using gel densitometry, 20 µL of the sixth day culture supernatant was loaded on 12% SDS-PAGE, resolved, and analyzed. The average of the total extracellular protein (Bradford assay) for each triplicate set was monitored throughout six days. HSA produced was quantified using gel densitometry.

Three parameters, namely, temperature, methanol level, and peptone concentration were selected for statistical optimization by CCD (Central Composite Design) software. In CCD, the lower and upper limit value for temperature was 20 and as 28 °C respectively, for methanol, it was set at 1% and 2% respectively. For peptone, these values were 3% and 4%. Three factorial level design was implemented with independent factors studied at three levels (−1, 0, and +1). For temperature, 17.27% and 30.73% were set as the axial values (out of the box) with central limit at 24 °C. Methanol was set at 0.66% (below the lower limit) and 2.34% (above the upper limit) with a central value of 1.5%. Similarly, peptone was 1.32% and 4.62% (below and above the set values of 2% and 4%), respectively, with a mid-value of 3%. The detailed chart is provided in Table S2 in the Supplementary file. A total of twenty experiments were set in triplicates. Cultivation conditions, sample withdrawal and assay conditions were the same as described above.

## Appendix B

Protocol for RNA isolation from *P. pastoris* followed by cDNA construction for qPCR

*Pichia* cells were harvested by centrifugation at 6000× *g* for 20 min followed by washing twice with RNase free DEPC treated water to remove the medium components. The media-free cells dissolved in minimum amount of DEPC water were then passed through a 5 mL syringe and snap frozen in liquid nitrogen forming white beads followed by storage at −80 °C for future use. Total RNA was extracted using TRIzol method (Sigma), where approximately 200 mg of beads were ground to powder with the help of liquid nitrogen in a DEPC treated pestle and mortar. The powder was transferred to a 1.5 mL MCT and the total volume was made to 1 mL with TRIzol reagent. Vigorous pipetting or vortexing was done to obtain a homogenous clear liquid which was further incubated for 5 min at room temperature to allow complete dissociation of the nucleoprotein complex. Sample was then centrifuged at highest rpm at 4 °C to remove insoluble debris and 200 µL of chloroform was added.

The total RNA was extracted by chloroform-isoamyl alcohol phase separation (lower red phase containing phenol chloroform, an interphase of protein followed by an upper aqueous phase of RNA) followed by addition of 500 µL of 2-propanol and finally ethanol precipitation to pellet the RNA. The final RNA pellet was dissolved in RNase free DEPC water. All experiments were carried out with DEPC treated plasticwares at 4 °C. A small aliquot of prepared RNA was run on 1% agarose gel (prepared in MOPS buffer) for quality assessment. Of the total RNA, 1 µg was converted to cDNA according to the iScript^TM^ cDNA Synthesis kit (Bio-Rad) protocol provided by the manufacturer using 1.0 µL Reverse Transcriptase enzyme and 5 µL iscript mix in a total volume of 20 µL. PCR amplification of the identified target genes was carried out in a 10 µL reaction mixture (3 µL of cDNA, 5 µL of 2 x Evagreen Supermix (Bio-Rad)), 1 µL of forward and reverse primer each in a total volume of 10 µL (made up with nuclease-free water)). The reaction was incubated at 58 °C in triplicates in a 96-well plate. The reported data are average of three technical replicates shown with standard deviation.

## Appendix C

Detailed description of Transcriptome sequencing

The libraries were sequenced on Illumina NextSeq 500 using 2*75 bp chemistry. PCR-enriched libraries were analyzed on 4200 Tape Station system (Agilent Technologies) using a high-sensitivity D1000 Screen tape as per the manufacturer’s protocol. High-quality reads (QV > 20) were obtained using Trimmomatic v0.35 and PE reads were considered for *de novo* assembly. The filtered high-quality reads were assembled into transcripts using Trinity-v 2.4.0 with a k-mer of 25. The transcripts were further clustered into Unigenes based on global sequence identity threshold of 90% using CD-HIT-EST v 4.6 to reduce redundancy. RSEMv 1.2.31 was used to compute abundances based on mapping of sample-wise HQ reads to the pooled Unigenes via Bowtie2v2.2.6. The predicted Unigenes were validated based on FPKM ≥ 1, i.e., the estimated FPKM should be ≥ 1 in at least one sample. The Transdecoder v2.0 was used to predict coding sequences from the Unigenes. The predicted Unigene and CDS sequences were searched against NCBI non redundant protein database (Nr) using Basic Local Alignment Search Tool (BLASTx) (E value: 1 × 10^−5^) to find identity to the nearest fungal species. Distribution of GO terms for both Unigenes and CDS across the categories-BP (Biological Process), MF (Molecular Function) and CC(Cellular Components) were obtained through WEGO portal (http://wego.genomics.org.cn/cgi-bin/wego/index.pI) and Blast2GO.

Differential gene expression (DGE) analysis was performed on the validated Unigenes common between optimized (O) and unoptimized conditions (U) by employing a negative binomial distribution model in DESeq package (v1.22.1-http://www.huber.embl.de/users/anders/DESeq/). Dispersion values were estimated with the following parameters: method = blind, sharing Mode = fit-only and fit-type = local, log_2_fold change (FC) value was calculated on the root mean square error (RSEM) computed expected counts using the formula:

FC = Log_2_ (Treated/Control)

The Unigenes having FC value greater than zero were considered as upregulated, whereas values less than zero were downregulated. A *p*-value threshold of 0.05 was used to filter statistically significant result.

A heat map was constructed using the log-transformed and normalized value of Unigenes based on Pearson uncentred correlation distance, as well as the complete linkage method. A Volcanic plot was constructed for differentially regulated genes, where *p*-value was <0.05 and log_2_fold change > 0.0 for upregulated genes indicated in red color and genes with *p*-value < 0.05 and log_2_fold change < 0.0 were indicated in green colur and grey color was used to show genes expressed under both the conditions.

## Appendix D

ELISA protocol

Hundred µL of standard/test sample was loaded in duplicates in the designated wells. The plate was covered and incubated at 20–25 °C for 1 h and then washed four times followed by addition of 100 µL of anti-albumin detection antibody to each well. The incubation and washing steps were repeated. Next, 100 µL of HRP Solution A was added to each well and subsequently incubated for 30 min and washed four times thoroughly. Hundred µL of TMB substrate was added and the plate was incubated in dark for 30 min. Finally, the reaction was stopped by adding 100 µL of the stop reaction to each well. The absorbance was measured at 450 nm in a plate reader.
