# Supplementary material for: Statistically Designed Medium Reveals Interactions between Metabolism and Genetic Information Processing for Production of Stable Human Serum Albumin in Pichia pastoris
Source: Biomolecules. 2019 Oct 4;9(10):568. doi: 10.3390/biom9100568 (PMC6843683; doi:10.3390/biom9100568)
Supplement: Supplementary file 1 [file biomolecules-09-00568-s001.pdf]

Statistically Designed Medium Reveals Interactions Between Metabolism and Genetic information Processing for Production of Stable Human Serum Albumin in *Pichia pastoris*

Nitu Maity and Saroj Mishra *

Department of Biochemical Engineering and Biotechnology, Indian Institute of Technology Delhi, Hauz-Khas, New-Delhi 110016; nitumaity@gmail.com

* Correspondence: saroj98@hotmail.com; Tel.: 91-011-2659-1007

Index

Figures **Pg 3-7**

1. Bioinformatics workflow **Pg 3**

Figure S1: Bioinformatics workflow Pg 3

2. Screening of 2-copy constructs for production of stable HSA **Pg 3**

Figure S2. SDS-PAGE analysis of culture filtrate produced by 2-copy constructs. (a) Lanes 1-6: Clone # s are shown above the lane numbers. Lane 7: 1-copy construct, Lane 8: Commercial HSA producing clone (com), Lane 9: commercial HSA, Lane 10: molecular weight ladder. (b) Lanes 1-6: Clone # s are shown above the lane numbers. Lane 7: 1-copy construct, Lane 8: Commercial HSA producing clone (com), Lane 9: molecular weight ladder, Lane 10: commercial HSA (c) Lanes 1-7: Clone # s are shown above the lane numbers. Lane 8: 1-copy construct, Lane 9: Commercial HSA producing clone (com), Lane 10: commercial HAS . Pg 3

3. Unigenes identified based on Gene Ontology **Pg 4**

Figure S3. (a) Classification of genes based on Gene Ontology, b) WEGO plot showing GO for Unigenes. Pg 4

4. CDS identified based on Gene Ontology **Pg 5**

Figure S4. (a) Classification of CDS transcripts base on gene ontology IDs, (b) WEGO plot showing GO for CDS sequences. Pg 5

5. **Volcanic plot representation of up- and down-regulated genes.** **Pg 6**

Figure S5. Volcanic plot representing differentially expressed genes. Pg 6

6. **Percent distribution of up- and down-regulated genes.** **Pg 7**

Figure S6. (a) Percentage distribution of up- and down-regulated genes (cut-off p values < 0.05) mapped to their respective GO biological pathways (KEGG pathways) (b) Venn diagram representing genes up- or down-regulated or expressed under both conditions. (c) List of exclusively up- and down-regulated genes. (d) Number of up-, down-regulated and genes expressed under both the conditions.

Pg 7

Tables **Pg 8-17**

Table S1 Design of Experiments (DOE) using Plackett-Burman methodology (unit for temperature is degrees Celsius, inoculum is O.D. units, where 1 $O.D_{600}$ unit corresponds to approximately 10^7cells/ml, sorbitol and peptone are in percentage (w/v), methanol in percentage (v/v). Pg 8

Table S2 Design of experiments (DOE) using Central Composite Design methodology (CCD).

Pg 9

Table S3 Codon usage table of cloned HSA Pg 10

Table S4 Regression statistics and ANOVA for Plackett-Burman design Pg 11

Table S5 Analysis of variance (ANOVA) for Quadratic model obtained from Face centered CCD
Pg 12

Table S6 KEGG pathway classification of genes transcribed under optimized and un-optimized conditions Pg 13

Table S7 A comparison of the up- and down- regulated genes obtained from studies involving methanol metabolism [54], temperature [65] and present study.

Pg 14

1. Bioinformatics Flowchart

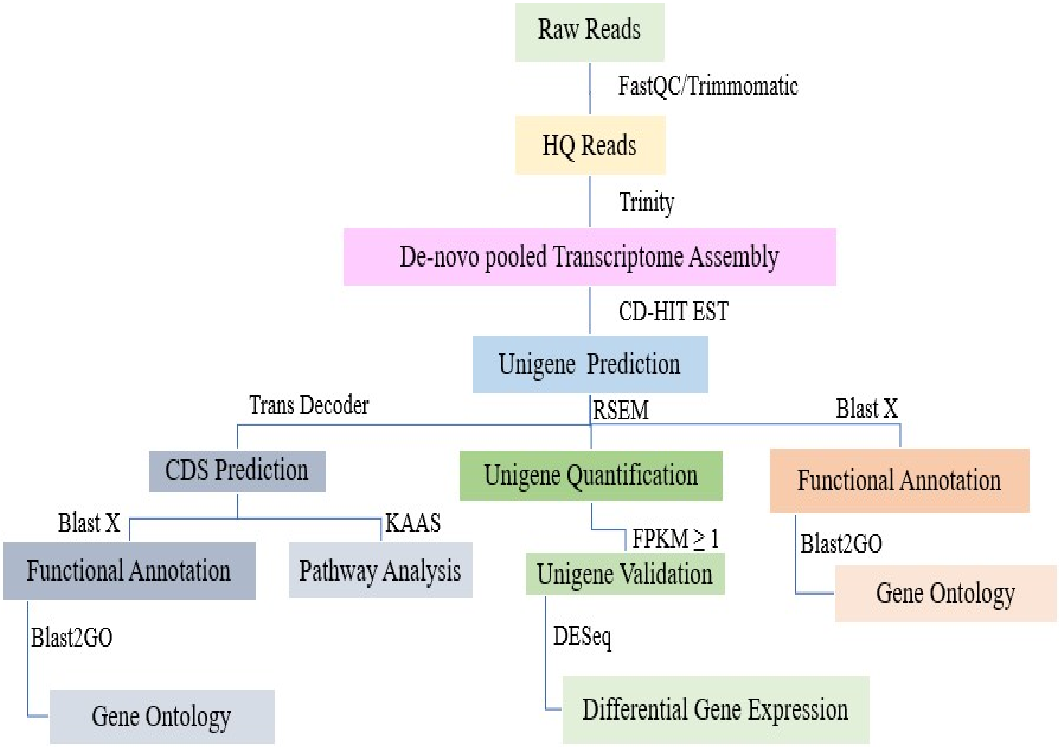

Figure S1. Bioinformatics workflow.

2. Screening of 2-copy constructs for production of stable HSA

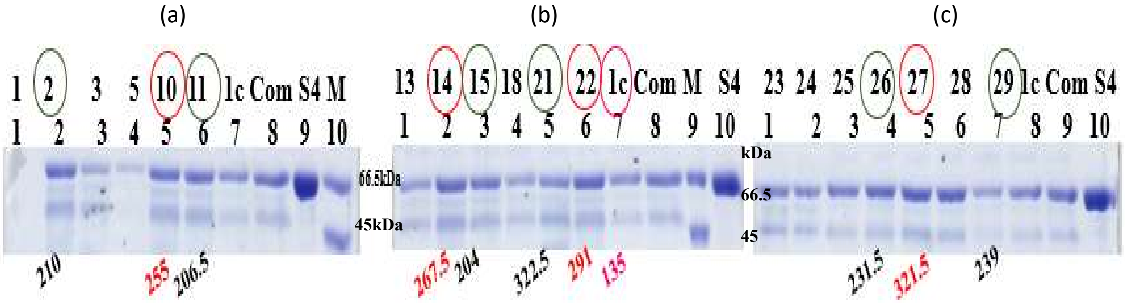

Figure S2. SDS-PAGE analysis of culture filtrate produced by 2-copy constructs. (a) Lanes 1-6: Clone # s are shown above the lane numbers. Lane 7: 1-copy construct, Lane 8: Commercial HSA producing clone (com), Lane 9: commercial HSA, Lane 10: molecular weight ladder. (b) Lanes 1-6: Clone # s are shown above the lane numbers. Lane 7: 1-copy construct, Lane 8: Commercial HSA producing clone (com), Lane 9: molecular weight ladder, Lane 10: Commercial HSA. (c) Lanes 1-7: Clone # s are shown above the lane numbers. Lane 8: 1-copy construct, Lane 9: Commercial HSA producing clone (com), Lane 10: Commercial HSA.

3. Unigenes identified based on Gene Ontology

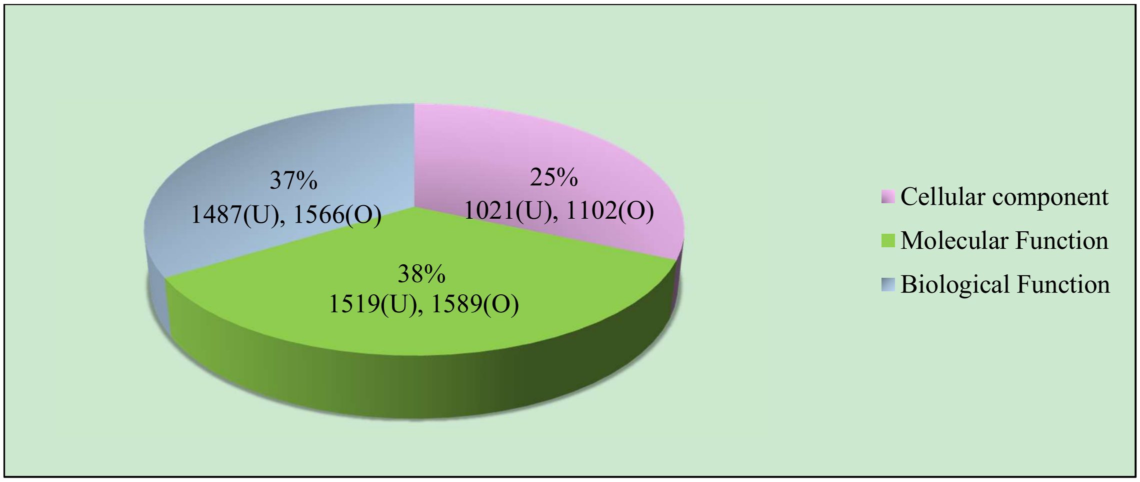

Figure S3. (a) Classification of genes based on Gene Ontology

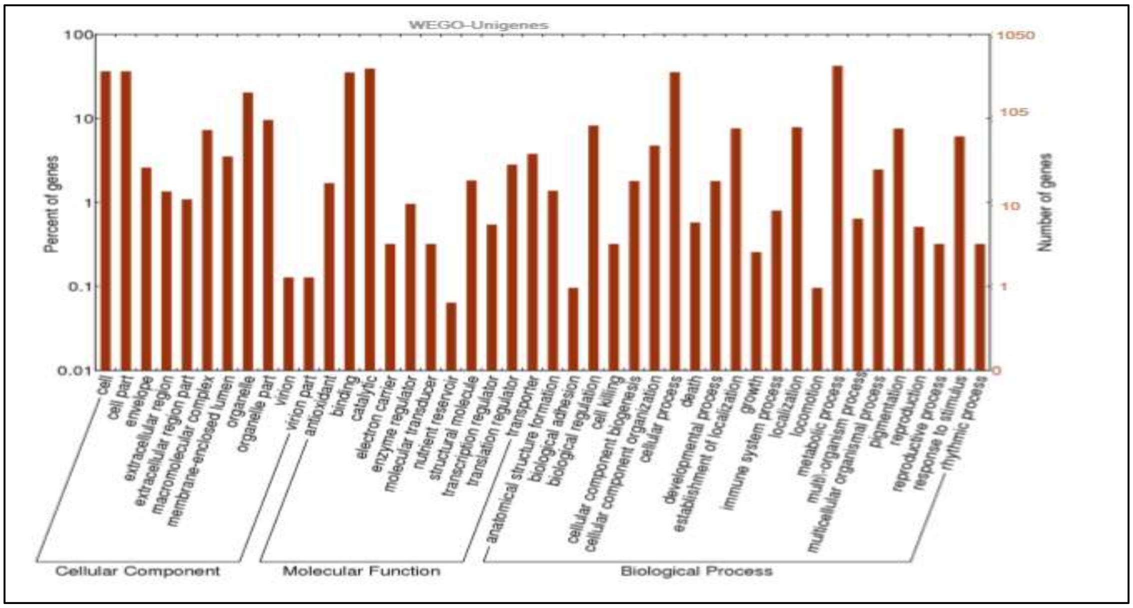

Figure S3. (b) WEGO plot showing GO for Unigenes.

4. CDS identified based on Gene Ontology

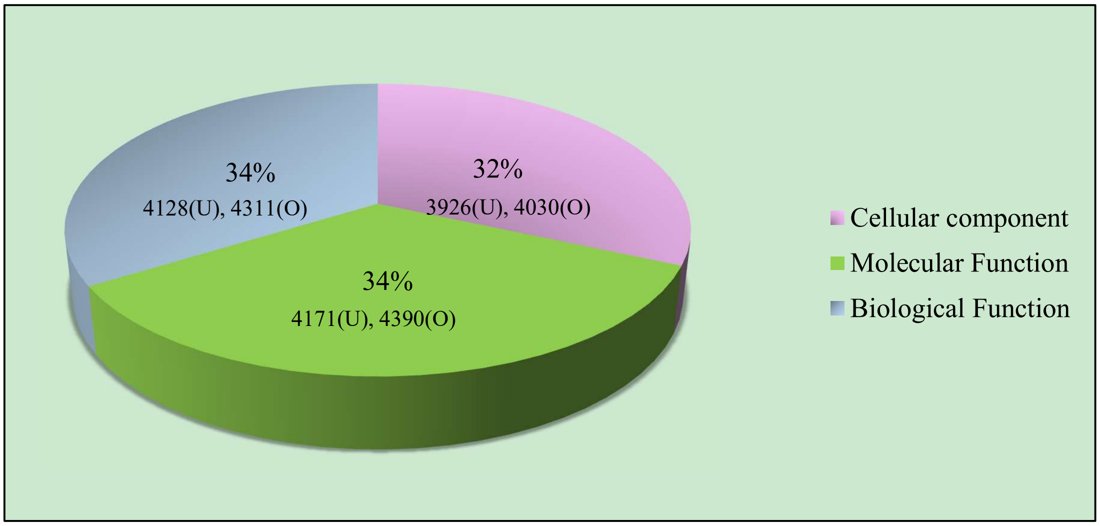

Figure S4. (a) Classification of CDS transcripts base on gene ontology GO IDs.

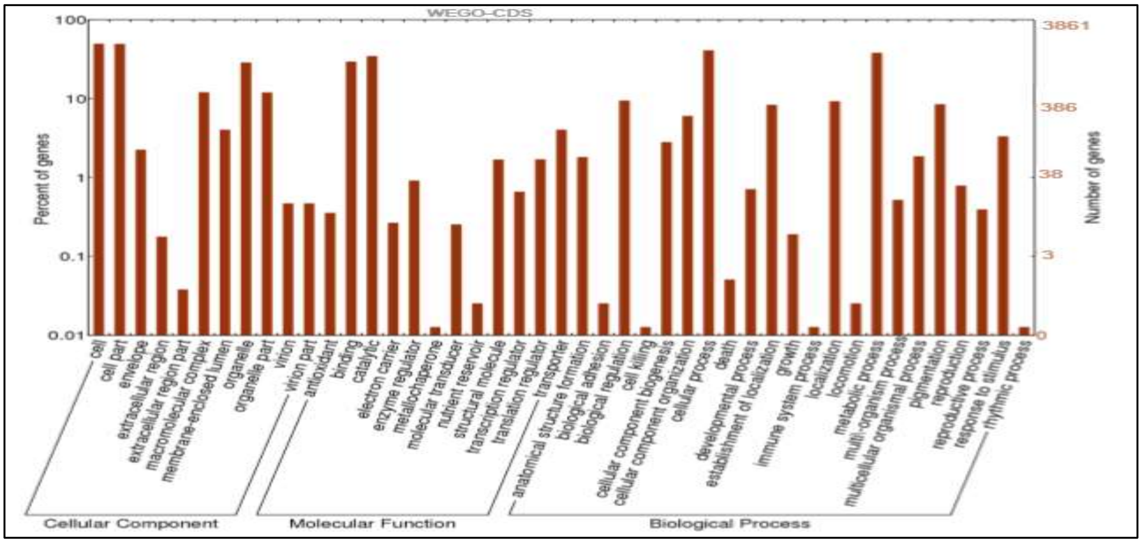

Figure S4. (b) WEGO plot showing GO for CDS sequences.

5. Volcanic plot representation of up- and down-regulated genes.

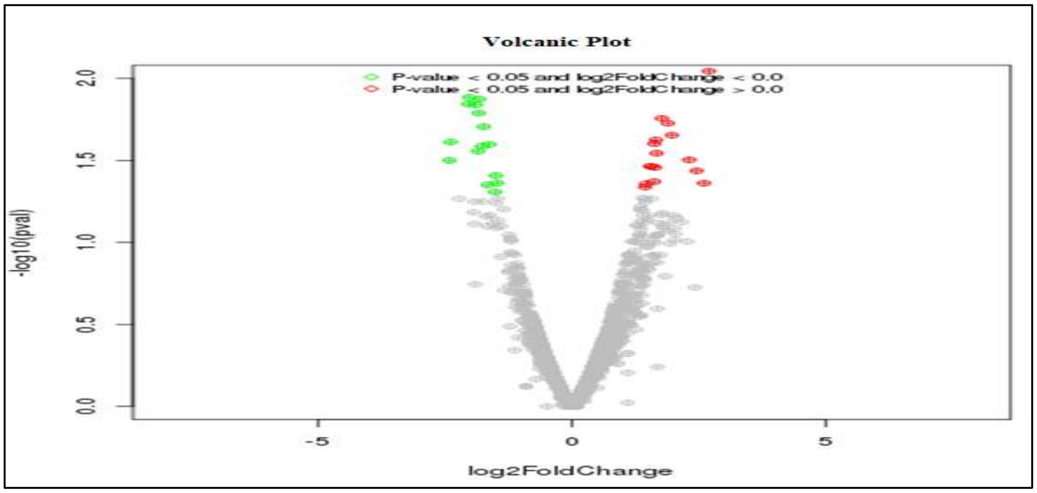

Figure S5. Volcanic plot representing differentially expressed genes.

6. Percentage distribution of up- and down-regulated genes.

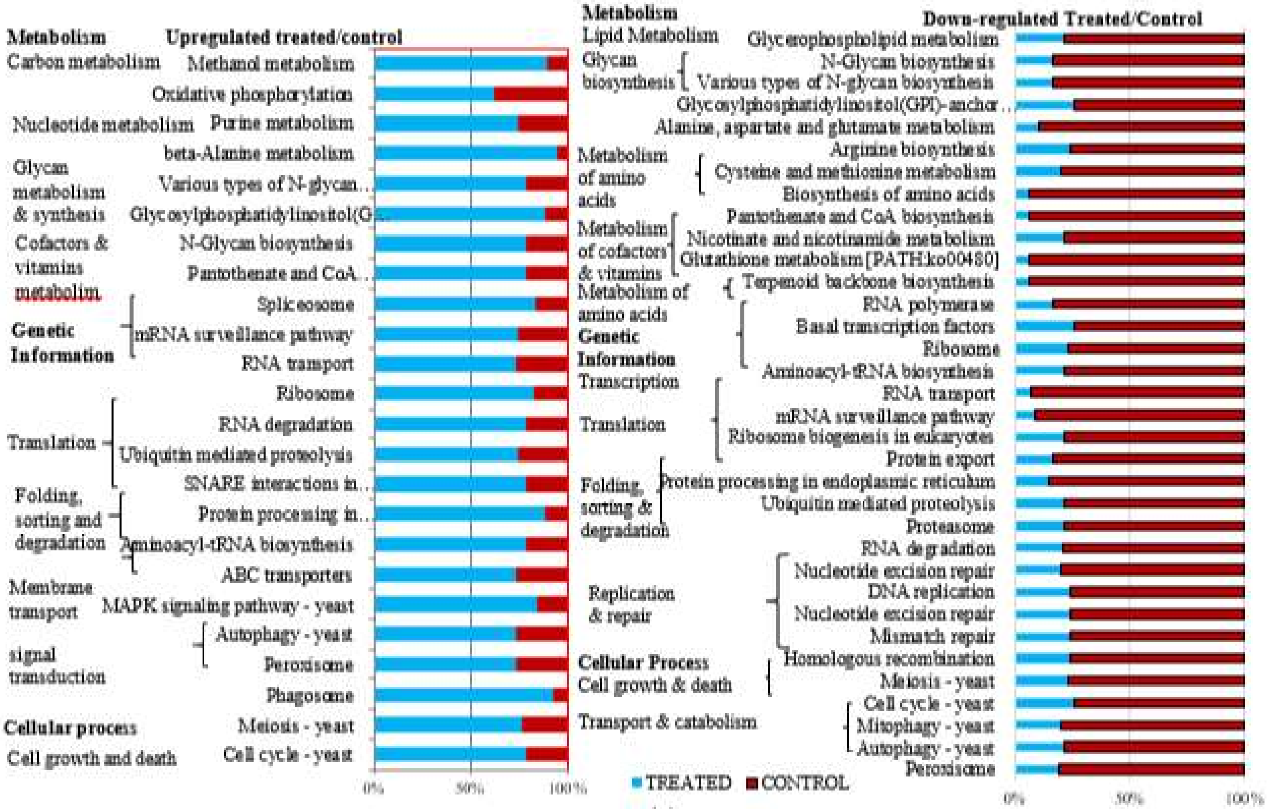

(a)

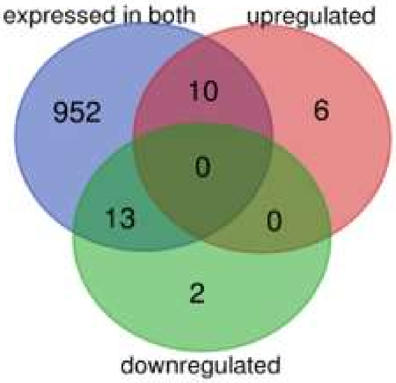

(b)

Exclusively upregulated	6	• Mitochondrial alcohol dehydrogenase isozyme III<br>• (hydroxymethyl)glutathione dehydrogenase<br>• Plasma membrane ATP-binding cassette (ABC) transporter required for the export of a- catalyze<br>• SEA (Seh1-associated) complex subunit<br>• Translation elongation factor EF-1 gamma<br>• Plasma membrane localized that protects membranes from desiccation
Exclusively downregulated	2	• Alternative oxidase<br>• Heat Shock Hsp90

(c)

List names	number of genes	number of unique genes
expressed in both	975	975
downregulated	15	15
upregulated	16	16
Overall number of unique genes		983

(d)

Figure S6. (a) Percentage distribution of up- and down-regulated genes (cut-off p values < 0.05) mapped to their respective GO biological pathways (KEGG pathways) (b) Venn diagram representing genes up- or down-regulated or expressed under both conditions. (c) List of exclusively up- and down-regulated genes. (d) Number of up-, down-regulated and genes expressed under both the conditions.

Table S2 Design of Experiments (DOE) using Plackett-Burman methodology (unit for temperature is degrees Celsius, inoculum is O.D. units, where 1 $O.D_{600}$ unit corresponds to approximately 10^7cells/ml, Sorbitol and peptone are in percentage (w/v), methanol in percentage (v/v).

Run	Temp (°C)	Inoculum	pH	Sorbitol (%)	MeOH (%)	RPM	Peptone (%)	Dummy variables				HSA produced (mg/L)	
												Actual	Predicted
1	22	10	7	0.5	2	250	4	-1	-1	-1	1	275±1.12	275.32
2	22	10	6	1	2	220	4	1	1	-1	-1	267±0.87	267.26
3	28	10	7	0.5	1	220	4	-1	1	1	-1	244±0.94	244.07
4	28	1	7	1	1	250	4	1	-1	-1	-1	243±1.31	243.42
5	22	1	6	1	1	250	4	-1	1	1	1	257±1.26	256.69
6	28	1	6	0.5	2	220	4	1	-1	1	1	258±0.95	258.49
7	28	10	6	1	2	250	2	-1	-1	1	-1	262±1.24	263.36
8	22	1	6	0.5	1	220	2	-1	-1	-1	-1	274±0.68	275.00
9	22	10	7	1	1	220	2	1	-1	1	1	270±0.78	270.49
10	28	1	7	1	2	220	2	-1	1	-1	1	272±1.38	271.64
11	28	10	6	0.5	1	250	2	1	1	-1	1	254±1.24	253.44
12	22	1	7	0.5	2	250	2	1	1	1	-1	293±1.38	292.98
% contribution of each factor	40.13	2.22	2.11	2.63	27.75	0.01	25.06						

Table S2 Design of Experiments (DOE) using Central Composite Design methodology (CCD).

Run	X_1: Temperature (°C)		X_2: Methanol (%)		X_3: Peptone (%)		**Y*** HSA produced (mg/L)	
	Coded	Actual	Coded	Actual	Coded	Actual	Predicted	Actual
1	0	24	0	1.5	0	3	344.99	336±0.93
2	-1	20	1	2	1	4	232.36	235±0.85
3	0	24	-1.68	0.66	0	3	265.22	268±0.96
4	1	28	-1	1	1	4	235.31	238±0.84
5	0	24	0	1.5	0	3	344.99	345±0.86
6	1	28	1	2	1	4	232.13	235±1.14
7	0	24	0	1.5	0	3	344.99	350±1.17
8	0	24	1.68	2.3	0	3	238.00	235±0.87
9	0	24	0	1.5	0	3	344.99	342±1.21
10	-1.68	17.3	0	1.5	0	3	300.15	303±1.02
11	1.68	30.7	0	1.5	0	3	261.07	258±1.24
12	-1	20	1	2	-1	2	293.53	291±1.11
13	0	24	0	1.5	-1.68	1.3	307.64	311±0.94
14	0	24	0	1.5	1.68	4.7	221.58	218±1.34
15	0	24	0	1.5	0	3	344.99	350±1.38
16	0	24	0	1.5	0	3	344.99	347±0.92
17	-1	20	-1	1	1	4	241.05	239±0.89
18	1	28	1	2	-1	2	252.80	255±1.62
19	-1	20	-1	1	-1	2	322.72	320±1.24
20	1	28	-1	1	-1	2	276.48	274±1.67

*Y: Response was measured in terms of production of HSA as observed by gel densitometry on SDS-PAGE.

Table S3 Codon usage table of cloned HSA

Codon	Number	/1000	Codon	Number	/1000	Codon	Number	/1000
GCG	1.00	1.48	ATA	1.00	1.48	AGG	3.00	4.44
GCA	20.00	29.59	ATT	6.00	8.88	AGA*	13.00	19.23
GCT	28.00	41.42	ATC	8.00	11.83	CGG	1.00	1.48
GCC	26.00	38.46				CGA	2.00	2.96
						CGT	5.00	7.40
						CGC	2.00	2.96
TGT	23.00	34.02	TTG	26.00	38.46	AGT	3.00	4.44
TGC	12.00	17.75	TTA	9.00	13.31	AGC	3.00	4.44
			CTA	5.00	7.40	TCG	1.00	1.48
			CTT	10.00	14.79	TCA	5.00	7.40
			CTC	3.00	4.44	TCT	12.00	17.75
			CTG	15.00	22.19	TCC	8.00	11.83
GAT	26.00	38.46	ATG	7.00	10.36	ACG	3.00	4.44
GAC	14.00	20.71				ACA	8.00	11.83
						ACT	15.00	22.19
						ACC	10.00	14.79
GAG	35.00	51.78	AAT	9.00	13.39	GTG	11.00	16.27
GAA	34.00	50.30	AAC	13.00	19.23	GTA	5.00	7.40
						GTT	18.00	26.63
						GTC	13.00	19.23
TTT	18.00	26.63	CCG	1.00	1.48	TGG	1.00	1.48
TTC	19.00	28.11	CCA	13.00	19.23			
			CCT	7.00	10.36			
			CCC	7.00	10.36			
GGG	2.00	2.96	CAG	9.00	13.31	TAT	10.00	14.79
GGA	7.00	10.36	CAA	12.00	17.75	TAC	9.00	13.31
GGT	4.00	5.92						
GGC	3.00	4.44						
CAT	10.00	14.79	AAG	29.00	42.90	TGA	0.00	0.00
CAC	8.00	11.83	AAA	32.00	47.34	TAG	1.00	1.48
						TAA	1.00	2.96

*Bias for certain codons is indicated in red font.

Table S4 Regression statistics and ANOVA for Plackett-Burman design

**Source**	**Sum of Squares**	**D*f***	**Mean Square**	**Coefficient estimate**	**Standardized Effect**	**% contribution**	***F*-value**	***t*-stat**	***p*-value**
Model/Intercept	2214.97	7	316.42	264.35			33.47		0.0022*
Temperature	889.76	1	889.76	-8.61	-17.22	40.13	123.37	40.2	0.0004*
Inoculum	49.09	1	49.09	-2.02	-4.045	2.22	0.8743	9.29	0.4027
pH	46.69	1	46.69	1.97	3.945	2.11	0.5390	9.12	0.5036
Sorbitol	58.30	1	58.30	-2.20	-4.41	2.63	5.35	9.89	0.0817
Methanol	615.33	1	615.33	7.16	14.32	27.75	55.82	32.1	0.0017*
RPM	0.2494	1	0.2494	-0.1442	-0.288	0.01	0.0943	1.02	0.7741
Peptone	555.56	1	555.56	-6.80	-13.61	25.06	48.26	31.8	0.0023*
Residual	2.18	4	0.5454						
Cor Total	2217.15	11							

D*f*: Degree of freedom, *F*: Fischer's function, *t*-stat: Student's t-test, *p* value: Level of significance**, *** refers to significant values

Fit Statistics

Std. Dev.	5.04	R^2	0.9940
Mean	287.50	Adjusted R^2	0.9886
C.V. %	1.75	Predicted R^2	0.9750
		Adeq Precision	34.6393

The correlation coefficient (R) is 0.996. The Predicted R^2 of 0.9750 is in reasonable agreement with the Adjusted R^2 of 0.9886; i.e. the difference is less than 0.2.

Table S5 Analysis of variance (ANOVA) for Quadratic model obtained from Face centered CCD

Source	Sum of Squares	D*f*	Mean Square	*F*-value	*p*-value
**Model**	41979.14	9	4664.35	183.74	< 0.0001*
X_1 - (Temperature, °C)	1843.73	1	1843.73	72.63	< 0.0001*
X_2 - (Methanol, % v/v)	894.06	1	894.06	35.22	0.0001*
X_3 – (Peptone, % w/v)	8939.47	1	8939.47	352.14	< 0.0001*
X_1X_2	15.13	1	15.13	0.5958	0.4580
X_1X_3	820.13	1	820.13	32.31	0.0002*
X_2X_3	210.13	1	210.13	8.28	0.0165*
X_1^2	7466.96	1	7466.96	294.14	< 0.0001*
X_2^2	15708.71	1	15708.71	618.79	< 0.0001*
X_3^2	11639.43	1	11639.43	458.50	< 0.0001*
**Residual**	253.86	10	25.39		
Lack of Fit	109.86	5	21.97	0.7629	0.6131
Pure Error	144.00	5	28.80		
**Cor Total**	42233.00	19			

*:Represents significant values, D*f*: Degree of freedom, *F*: Fischer's function, *p*: Level of significance.
The model *F* value of 183.74 implies that the model is significant. There is only 0.01% chance that an *F*-value so large could occur due to noise.
P value less than 0.05 indicate that the model terms are significant. Thus, all individual parameters and interactions, other than between temperature and methanol, are significant.

Table S6 KEGG pathway classification of genes transcribed under optimized and un-optimized conditions

	Optimized	Un-optimized
**Metabolism**		
Carbohydrate metabolism	251	250
Energy metabolism	181	181
Lipid metabolism	151	148
Nucleotide metabolism	151	147
Amino acid metabolism	250	253
Metabolism of other amino acids	78	77
Glycan biosynthesis and metabolism	93	91
Metabolism of cofactors and vitamins	184	185
Metabolism of terpenoids and polyketides	38	38
Biosynthesis of other secondary metabolites	41	41
Xenobiotics biodegradation and metabolism	35	35
**Genetic Information Processing**		
Transcription	186	185
Translation	404	404
Folding, sorting and degradation	350	345
Replication and repair	132	133
**Environmental Information Processing**		
Membrane transport	16	16
Signal transduction	346	345
**Cellular Processes**		
Transport and catabolism	374	371
Cell growth and death	264	261

Table S7 A comparison of the up- and down- regulated genes obtained from studies involving methanol metabolism [54], temperature [65] and present study.

		METHANOL [54]	TEMPERATURE [65]	OPTIMIZED/UN-OPTIMIZED MEDIUM (Present study)
**METABOLIC PATHWAYS**	**GENE NAME (Function)**	**LOG_2FC**	**LOG_2FC**	**LOG_2FC**
**Methanol metabolism**	AOX2: Alcohol oxidase1	3.5	-0.82	3.5
	AOX1 :Alcohol Oxidase	5.32	-	1.09
	FLD: Flavodoxin reductase	2.87	-	-
	FGH: S-formyl glutathione	3.46	-	0.85
	FDH: Formate dehydrogenase	3.33	-	1.13
	CAT: Catalase1	1.88	-	2.02
	DAS: Dihydroxy acetone synthase	6.68	-	1.25
	FLD1: S-hydroxymethyl glutathione dehydrogenase	-	-	1.5
	DAK: dihydroxyacetone kinase	2.79	-	2.46
	FBP; Fructose1,6 bisphosphate	1.81	-	1.17
	MOX: Methanol oxidase	-	-	2.34
**Energy metabolism**	ACO1: Aconitate hydratase	-	-0.45	-
	ACS1: Acetyl CoA synthetase	-	-0.51	-1.12
	ALD4: Aldehyde dehydrogenase 1	-	-0.45	4.08
	CIT1: Citrate synthase	-	-0.92	1.09
**Glycolysis**	HEX: Hexokinase	-	-	1.17
	ADHP: Alcohol dehydrogenase	-	-	1.23
**Pyruvate metabolism**	ALDH: aldehyde dehydrogenase	-	-	4.08
**Fatty acid Biosynthesis**	ACAA1: Acetyl CoA-Acyltransferase 1	-	-	1.78
	FUM1: Fumarase	-	-0.42	-
	ICL1: Isocitrate lyase	-	-0.42	-0.37
	MDH1: Malate dehydrogenase	-	-0.60	-0.94
	LPD1: Dihydrolipoamide dehydrogenase	-	-0.62	-
**Glycerol metabolism**	GUT1: Glycerol kinase1	-3.8	0.47	-1.17
	GUT2 :Glycerol kinase	-2.74	-	-1.25
	STL1: Glucose inactivated glycerol proton symporter STL1	-0.99	-	-0.13
**Oxidative stress**	CCP1:Cytochrome C peroxidase	-1.81	0.79	-
	CAT1:Catalase 1	-3.33	1.88	-
	YFL054C: aquaglyceroporin related protein	-2.99	-	-

**PEROXISOME BIOSYNTHESIS**	PEX1: Peroxin 1	1.15	-	1.2
	PEX2: Peroxin 2	2.63	-	-
	PEX3: Peroxin 3	1.4	-	-
	PEX4: Peroxin 4	0.63	-	1.75
	PEX5: Peroxin 5	2.88	-	-
	PEX6: Peroxin 6	2.15	-	-
	PEX7: Peroxin 7	-	-	-
	PEX8: Peroxin 8	1.64	-	-
	PEX11: Peroxin 11	3.58	-	-
	PEX12: Peroxin 12	2.19	-	-
	PEX13: Peroxin 13	2.54	-	-
	PEX14: Peroxin 14	1.51	-	-
	PEX19: Peroxin 15	0.86	-	-
	PEX25: Peroxin 25	0.93	-	-
	PEX28: Peroxin 28	-	-	-
	PEX29: Peroxin 29	-	-	-1.07
	PXA: peroxisomal long chain fatty acid import protein	-	-	1.09
**Nitrogen metabolism**	GS: Glutamine synthetase	-	-	1.43
	GOGAT: Glutamate synthetase	-	-	1.23
**Amino acid metabolism**	AGX1: Ala-glyoxylate amino transferase	-	-0.58	-
	GDH1: Glutamate dehydrogenase #1	-	0.73	1.14
	GDH2: Glutamate dehydrogenase #2	-	0.72	1.29
	ILV5: Aceto-hydroxy acid reducto-merase	-	-0.38	-
	MET6: Homocysteine methyltransferase 1	-	0.66	0.57
	MET6: Homocysteine methyltransferase 2	-	0.66	0.47
	SAM2: S-adenosyl methionine synthetase	-	-0.32	-
**TRANSCRIPTIONAL/TRANSLATIONAL REGULATION**	GAT1: Solute carrier family 6 member 1	-1.05	-	0.65
	HAA1: Transcriptional activator 1	-0.76	-	-0.66
	RTG1: Retrograde regulator protein1	-1.14	-	-0.33
	PHO2: Phosphate transporter regulatory protein	-1.37	-	-0.77
	CST6: ATF/CREB activator 1	-1.09	-	

	EIF3J: Translation initiation factor 3 of subunit J	-	-	-4.08
	EIF4G: Translation initiation factor 4G	-	-	-2.56
	SRP68: signal recognition particle subunit	-	-	-1.89
	EIF4A3: ATP-dependent RNA helicase	-	-	1.08
**RIBOSOME/RNA BIOGENESIS**	GSP1: GTP binding nuclear protein	-	0.32	-
**PROTEIN FOLDING/GENE EXPRESSION**	HSP31 :Chaperone	-	-0.55	-
	HSP60: Chaperone	-	-0.40	-
	HSP82: Chaperone	-	-0.46	-
	HSP90A: Chaperone	-	-	-2.05
	KAR2ER: ATPase BiP	-	-0.37	-0.27
	PDI1: Protein disulphide isomerase	-	0.35	0.44
	SSA4 : Secretion helper (SH)	-	-1.40	-0.94
	SSA4: SH 50kDa	-	0.37	-
	SSA4: SH 25kDa	-	0.74	-
	SSB1: Single strand DNA-binding protein	-	-0.99	-
	SSB1: (50kDa)	-	0.80	-
	SSB1: (25kDa)	-	0.77	-
	SEC 20: Transport protein	-	-	2.75
	SEC 23: Transport protein	-	-	3.2
	SEC 62: Translocation protein	-	-	-2.45
	ALG13: β-,4,-N acetyl glucosaminyl transferase	-	-	2.56
	ALG14: β-,4,-N acetyl glucosaminyl transferase	-	-	-1.23
	GPI7: Ethanolamine phosphotransferase	-	-	-1.49
**UBIQUITIN PROTEASOME SYSTEM**	LAP4 : metallo-amino peptidase APE1	1.7	-	-1.07
	PEP4: proteinase A	1.26	-	-
	PRB1: proteinase B	0.86	-	-
	YPS2: N-acetyl glucosamine kinase	2.2	-	-
	YPS2: : N-acetyl glucosamine kinase	0.91	-	-
	YPS3: Prophage ps3 protein01-like protein	0.72	-	-
	CDC23: Anaphase promoting complex	-	-	1.5

	YPS7: Aspartic proteinase Yapsin 7	0.6	-	-0.35
	SYVN1, HRD1: Ubiquitin protein ligase synoviolin			-1.13
	MKC7: Aspartyl protease	-	-	-0.58

Red refer to up-regulated genes in the optimized medium while green refer to down-regulated genes
